# Overcoming the Solubility Barrier of Ibuprofen by the Rational Process Design of a Nanocrystal Formulation

**DOI:** 10.3390/pharmaceutics12100969

**Published:** 2020-10-14

**Authors:** Andreas Ouranidis, Nikos Gkampelis, Elisavet Vardaka, Anna Karagianni, Dimitrios Tsiptsios, Ioannis Nikolakakis, Kyriakos Kachrimanis

**Affiliations:** 1 Department of Pharmaceutical Technology, School of Pharmacy, Aristotle University of Thessaloniki, 54124 Thessaloniki, Greece; ngkampel@pharm.auth.gr (N.G.); evardaka@pharm.auth.gr (E.V.); karagiak@pharm.auth.gr (A.K.); yannikos@pharm.auth.gr (I.N.); 2 Department of Chemical Engineering, Aristotle University of Thessaloniki, 54124 Thessaloniki, Greece; 3 Department of Neurophysiology, South Tyneside & Sunderland NHS Foundation Trust, Sunderland SR47TP, UK; dimitrios.tsiptsios@nhs.net

**Keywords:** nanocrystals, wet media milling, energy frameworks, slip planes, surface adsorption, nanosuspension stabilizer, solubility, molecular dynamics, scale up, Ibuprofen

## Abstract

Wet media milling, coupled with spay drying, is a commonly proposed formulation strategy for the production and solidification of nanosuspensions in order to overcome the solubility barrier of BCS Class II substances. However, the application of mechanically and thermally intensive processes is not straightforward in the cases of ductile and/or low melting point substances that may additionally be susceptible to eutectic formation. Using ibuprofen (IBU) as a model drug with non-favorable mechanical and melting properties, we attempt to rationalize nanocrystal formulation and manufacturing in an integrated approach by implementing Quality by Design (QbD) methodology, particle informatics techniques and computationally assisted process design. Wet media milling was performed in the presence of different stabilizers and co-milling agents, and the nanosuspensions were solidified by spray-drying. The effects of key process parameters (bead diameter, milling time and rotational speed) and formulation variables (stabilizer type and drug/stabilizer ratio) on the critical quality attributes (CQAs), i.e., Z-average size, polydispersity index (PDI), ζ-potential and redispersibility of spray-dried nanosuspensions were evaluated, while possible correlations between IBU free surface energy and stabilizer effectiveness were studied. The fracture mechanism and surface stabilization of IBU were investigated by computer simulation of the molecular interactions at the crystal lattice level. As a further step, process design accounting for mass-energy balances and predictive thermodynamic models were constructed to scale-up and optimize the design space. Contemplating several limitations, our multilevel approach offers insights on the mechanistic pathway applicable to the substances featuring thermosensitivity and eutectic tendency.

## 1. Introduction

A significant proportion, about four out of ten of under-development novel active pharmaceutical ingredients (APIs) intended for per os use, fall under the Class II of the Biopharmaceutics Classification System (BCS) [1]. The dual prevalence amongst them of poor solubility/dissolution rate and high cell membrane permeability, stipulates the limiting factor of GI absorption after oral delivery [2]. Increasing their aqueous solubility/dissolution profile is a challenging task that may be addressed by expanding the specific surface area available for contact with the biological fluids i.e., by particle size reduction [3]. The enhanced saturation solubility occurrence of ultra-fine powders was explained and quantified by the Kelvin and Ostwald–Freundlich equations, respectively.

Reasonably, nano-comminution of BCS II class substances, ascends as an enabling formulation tool, linked to improved drugability owing to favorable absorption and absolute bioavailability pharmacometrics. Wet media milling (WMM) has been proposed as an industrially feasible comminution process achieving API particle diameter below the 1000 nm scale [4], delivering nanocrystal dispersions conveniently referred to hereafter in this context as nanosuspensions [5]. Several nanosuspensions produced by WMM, such as Rapamune^®^ (Pfizer (Wyeth), New York City, NY, USA), Emend^®^ (Merck, Kenilworth, NJ, USA), Tricor^®^, Lipanthyl^®^ (Abbott Laboratories, Fournier Pharma, Montréal, QC, Canada), Megace^®^ ES (PAR Pharmaceuticals, Woodcliff Lake, NJ, USA), and Invega^®^, Sustenna^®^, Xeplion^®^ (Janssen, Beerse, Belgium), received marketing authorization, fueling the research interest of academia and industry.

Nanosuspension production is employing a variety of WMM techniques and instrumentation, (stirred media and vibration mills, etc.), among which planetary ball mills are the most energy-intensive [6,7]. The operating principle of a planetary ball mill refers to a grinding jar or pot positioned eccentrically on a sun wheel, while the two parts are set in motion in opposite directions, in a chosen ratio, usually 1:2, respectively. Beads contained inside the jar are subjected to superimposed movements of counter-linear rotations, termed Coriolis forces. The rotational velocity vector change generates frictional and impact forces between the grinding jar and beads, caused by random collisions between them and the solid material, inducing turbulent streams through the nanosuspension [8]. The shear stress applied on preexisting defects and cracks at the surface of the solid material, promotes crack propagation that results in fracture. This process, in turn, creates new interfaces, inextricably increasing the particle Gibbs free surface energy. As a result, nanosuspensions become thermodynamically unstable, nurturing Ostwald ripening as well as agglomeration, in order to compensate for the interfacial tension abatement [9].

The energy gradient directed agglomeration may be ameliorated by steric and/or electrostatic stabilization, contributed by the addition of non-ionic and/or ionic surfactants or other polymeric additives. The latter act as stabilizing agents by improving the wettability of the formed hydrophobic surfaces of the nanoparticles, thus increasing the activation threshold of the agglomeration event. Several additives, e.g., PVA, PVP, polysorbates, Poloxamers, and Solutol, have been utilized to stabilize various nanocrystalline particles [10]. Stabilizers are also thought to induce the so called adsorption induced reduction of strength of solids (AIRS), also known as the Rehbinder effect [11].

Nonetheless, the physical instability of nanosuspensions is of a thermodynamic nature and therefore may only become kinetically hindered, i.e., eventually the suspended particles undergo sedimentation, agglomeration and/or Ostwald ripening. Idcirco drying processes, most favorably spray drying (SD) or lyophilization, are performed following WMM, in order to enhance both the thermodynamic stability and processibility of the material for the final formulation steps, i.e., tableting or capsule filling. Spray dryers disperse and atomize the nanosuspensions to droplets into a pre-heated gas stream that facilitates the evaporation of the liquid phase. The state of atomization increases the surface area available for mass and heat transfer, thus allowing for lower operating temperatures in comparison to convective drying methods [12]. However, SD may itself effect aggregation and drug degradation when eutectic melting occurs. Certain formulations appear to form eutectic compositions, which exhibit melting point depressions, rendering the material incompatible with the thermal processing, thus impairing product stability and dissolution rate [13].

Ibuprofen (IBU), i.e., 2-(4-Isobutylphenyl) propanoic acid, is a nonsteroidal anti-inflammatory (NSAID) API classified as BCS II, exhibiting inconvenient viscoelastic properties such as strain rate sensitivity and brittle-ductile transition size limit of 854 μm [14]. Below this particle size, IBU crystals tend to flow rather than fragment, rendering further comminution by milling a nontrivial process [15]. Besides the poor grindability, attributed to ductility, the low melting point of IBU poses additional manipulation challenges, hence the thermal transfer through WMM and SD processing may result in the partial melting of the particles, fostering aggregation and/or amorphisation [16,17]. In addition, although the said melting point of IBU is approximately 76 °C, formulations containing IBU and terfenadine are reported to melt at temperatures ranging from 50 to 76 °C [18]. This melting point depression is attributed to the tendency of IBU to form eutectic compositions, as has also been found for IBU mixtures with diphenhydramine hydrochloride, astemizole and stearic acid [19]. These compositions exhibit structural deteriorations such as crumbling, pitting and fissuring, as well as decolorization pertaining to the rise of instability and storage issues [18].

Several successful attempts of IBU nanocomminution have been documented so far, including the production of IBU nanocrystals of 270 nm average diameter [14], composite amorphous IBU nanoparticles of 40 nm and 100 nm diameter prepared by SD in a lactose matrix and by encasing in silica shell, respectively, pursuing the dissolution enhancement objective [15]. Wet milling techniques versus dry milling of IBU have been evaluated, demonstrating the superiority of WMM resulting in larger surface area and improved dissolution rate of nanosuspensions under similar operating settings [15], while the preparation of inhalable fine particle fraction IBU agglomerates has also been reported as utilizing WMM followed by SD, achieving a mean particle diameter of 2–3 μm [16]. To our knowledge, no systematic study has been conducted so far that delves into the effect of key process parameters and formulation factors [10] on the IBU nanosuspension’s CQAs, when SD needs to be performed to WMM temperatures approximating melting and/or the possible eutectic point. Moreover, scalability and process performance design retaining wanted CQAs such as particle size, homogeneity and stability, remains unexplored.

In the present study, we propose an integrated approach that guides the formulation design from the bench to the manufacturing scale, aiming to develop IBU formulations capable of overcoming the solubility/dissolution barrier. We attempt to develop QbD-compliant optimization protocols of IBU nanocrystal formulations, processed by WMM and SD at temperature conditions adjacent to the melting point, combating IBU’s unfavorable viscoelastic behavior. In this perspective, we set out to investigate the effects of process variables, namely the amount and size of milling beads, milling speed and time, SD conditions, and formulation factors, such as the type and amount of stabilizer and co-milling agents, on the CQAs (Z-average diameter, ζ-potential, and redispersibility) of the solidified nanosuspensions. Concomitantly, we utilize molecular and first principle modeling computation at multiple levels of physical–chemical theory, in order to rationalize our experimental observations.

## 2. Materials and Methods

### 2.1. Materials

IBU (Boots, Nottingham, UK), which was kindly donated by VIANEX S.A. (Athens, Greece), was used as a model, poorly soluble API.

Lutrol F68 and Soluplus purchased from BASF, Germany, Natrosol 250 purchased from Ashland, USA, Methocel K4M purchased from Dow, USA, HPC-SL and HPC-SSL purchased from Nisso HPC, Japan and Pharmacoat 603, 606, 615 purchased from Shin-Etsu, Japan were employed as suspension stabilizers.

Mannitol (Pearlitol^®®^ 160C) purchased from Roquette Frères, (Lestrem, France) and Aerosil purchased from Evonik Industries AG, (Essen, Germany), were investigated as co-milling agents, and mannitol was employed as SD diluent and dispersibility-enhancing matrix former.

Distilled water was used as a dispersion medium for the wet milling process.

### 2.2. Methods

#### 2.2.1. Wet Media Milling

Preliminary wet media milling runs in order to select the most suitable stabilizer, and to screen the effects of bead diameter and stabilizer solution viscosity, were performed on a laboratory scale planetary ball mill (Pulverisette 7 Premium, Fritsch GmbH, Idar-Oberstein, Germany). 0.5 g of IBU crystals, 70 g of zirconium oxide milling beads, and 0.05 g of stabilizer (10% *w/w* relative to IBU amount) dissolved in 10 mL of distilled water, were added into a 45 mL milling jar and milling was effected at 450 rpm in 20 cycles of 3 min with 5 min breaks, to prevent the development of high temperature.

In order to select the most appropriate stabilizer, IBU was milled using the experimental settings described above, in the presence of different polymers, namely Lutrol F68, Soluplus, Natrosol 250, Methocel K4M, HPC-SL, HPC-SSL and Pharmacoat 603, 606, and 615. Aliquots of nanosuspension were withdrawn from the milling jar after 3, 6, 9, 15, 30 and 60 min of processing in order to determine the time evolution of IBU’s particle size in the presence of each stabilizer.

In order to evaluate the effect of stabilizer solution viscosity independently of the stabilizer type, three different Pharmacoat grades, (603, 606, and 615) with 0.8% *w/v* solution viscosities of 1.71, 2.0, and 2.25 mPa/s, respectively [20,21]. In this way, the effect of viscosity on the WMBM process was distinguished from all other possible effects due to the chemical substitution groups of cellulose.

The effect of bead diameter on the nanocrystal size was tested by repeating the milling process in the presence of Pharmacoat 603, using the beads of three different diameters, namely 0.3, 0.5, and 1.0 mm.

#### 2.2.2. Selection of Diluent/Co-Milling Agent

After selection of the optimal stabilizer, mannitol and Aerosil were evaluated as potential diluent agents on the basis of possible intermolecular interactions with IBU. The diluents were added at ratios of 0.5:1, 1:1 and 2:1 relative to IBU mass. The mixtures were subjected to Spray Drying at 85 °C on a Büchi B-191 Mini Spray dryer (Büchi, Switzerland) set at the following process parameters; air flow rate 800 m^3^/h, aspirator 80%, and pump speed 5%. Subsequently, the spray dried material was subjected to ATR-FTIR spectroscopy in the range of 600–4000 cm^−1^ with a resolution of 4 cm^−1^, after appropriate background subtraction, on an IR-Prestige-21 FT-IR spectrometer (Shimadzu Corporation, Kyoto, Japan), mounted with a horizontal Golden-Gate MKII single reflection ATR system (Specac, Kent, UK) equipped with ZnSe lenses. The number of scans per spectrum was set to *n* = 32. The spectra of the spray dried samples were compared to those of the physical mixtures prior to spray drying.

#### 2.2.3. Particle Size Determination

The particle size distribution of IBU was monitored during milling at predetermined time intervals (3, 6, 9, 15, 30 and 60 min), as zeta average (z-average) size and polydispersity index (PDI) determined by dynamic light scattering on a Zetasizer nano ZS instrument (Malvern Instruments, UK).

Crystals falling to particle sizes outside of the DLS operating range, as was the case for certain samples during the redispersibility testing, were measured by optical microscopy. An appropriate amount of the crystals was transported on a slide and dispersed in drops of silicone oil. The lens was then focused on a representative area of the sample and the size was determined by the automatic image analysis method, on a Laborlux S microscope (Leitz, Wetzlar, Germany), connected to a personal computer using the Quantimet 500 software (Leica, Cambridge, UK).

#### 2.2.4. Wettability Effect on the Size of Nanocrystals

The wettability of the IBU by stabilizer solutions was determined by the sessile drop method. Aqueous solutions of stabilizers (HPC-SL, HPC-SSL, Lutrol, Methocel K4M, Pharmacoat 603, Pharmacoat 606, and Pharmacoat 615) at the concentration used in the milling experiments (10% *w*/*w* of solids, which corresponds to 0.5% *w/v* aqueous solution) and IBU compressed tablets were prepared at maximum attainable relative density, on a manual hydraulic press. A single drop of stabilizer solution was dripped on the tablet surface and the contact angle formed between the solution droplet and the flat surface of the compressed tablet was monitored with the use of a digital camera, and measured immediately after application of the droplet and after 1 min, utilizing the add-in program drop analysis-lb-adsa Fiji (http://fiji.sc/, http://www.epfl.ch/demo/dropanalysis). The same procedure was followed to investigate the free surface energy of IBU with each different preselected stabilizer. Tablets were again prepared and the standard contact angle was measured with a droplet of water (W) and diiodomethane (D). The results were carried out by implementing the Owens-Wendt equation [22]:

(1) $${\left(\gamma_{s}^{d}\gamma_{l}^{d}\right)}^{0.5}+{\left(\gamma_{s}^{p}\gamma_{l}^{p}\right)}^{0.5}=0.5\gamma_{l}\left(1+\cos\Theta \right)$$

where γ_S_, γ_S_^d^ and γ_S_^p^ are the calculated values of surface free energy (SFE) and its dispersion and polar components of the solid, respectively; γ_l_, γ_l_^d^ and γ_l_^p^ are the known values of SFE and its polar and dispersive components of a measuring liquid, respectively (the l pointer referring to W or D); Θ is the contact angle between solid and liquid. Liquids with known parameters [22], like water (γ_l_ = 72.8, γ_l_^d^ = 21.8, and γ_l_^p^ = 51.0 mJ/m^2^) and diiodomethane (γ_l_ = 50.8, γ_l_^d^ = 49.5, and γ_l_^p^ = 1.3 mJ/m^2^) have been used.

#### 2.2.5. Solidification of Nanosuspensions by SD

Following milling, the nanosuspensions were subjected to SD on a Büchi B-191 Mini Spray dryer (Büchi, Switzerland) set at the following process parameters; air flow rate 800 m^3^/h, aspirator 80%, pump speed 5%. In order to study the effect of drying temperature in relation to the API’s melting point, the inlet air temperature was set to ±12% (±9 °C) of the melting point (76 °C), corresponding to 67 °C and 85 °C, respectively. Spray dried samples were stored in a desiccator over phosphorus pentoxide until further analysis.

#### 2.2.6. Differential Scanning Calorimetry (DSC)

In order to elucidate the possible effects of the stabilizer on the melting of IBU, DSC measurements were conducted on samples of drug coated with the best performing stabilizer by solvent drop grinding, and compared to an unprocessed physical mixture in terms of the melting point (depression). The samples were stored above phosporus pentoxide until constant weight was achieved and accurately weighted amounts (5 mg ± 0.2 mg) were placed in perforated aluminum pans and scanned between 25 °C to 120 °C at a heating rate of 10 °C/min. A constant nitrogen flow of 50 mL/min was maintained to provide a constant thermal blanket within the DSC cell.

#### 2.2.7. Determination of Redispersibility

Each dried sample was evaluated for its redispersibility, i.e., approximately 2–3 mg of solidified nanosuspension were immersed in 4 mL of distilled water and sonicated for 20 s, before determining their particle size by DLS or optical microscopy (depending on size range). The redispersibility index, RDI, was estimated for each run according to [23], by the following equation:

(2) $$\mathrm{RDI}=\frac{D_{o}}{D}$$

where D_o_ is the z-average diameter prior to SD, and D is the corresponding value of redispersed nanoparticles. RDI values close to unity indicate that the nanosuspension recovers its original particle size when dispersed in an aqueous medium.

#### 2.2.8. Optimization of WMM by Statistical Design of Experiment (DoE)

The amount of IBU was kept constant at 1 gr, HPMC Pharmacoat 603 was used as a stabilizer and mannitol as a co-milling agent. Milling was performed using 70 g of beads of 0.5 mm diameter. The rotation speed of the grinding chamber was set to 450 rpm, and grinding was performed at a total of 60 min, in 20 grinding cycles of 3 min each, followed by 5 min breaks, alternating the rotational direction after each milling cycle. Samples were collected after 3, 6, 9, 15, 30, 45 and 60 min and were exposed to the DLS measurement in order to document the process of the API’s comminution.

The design of experiment for the optimization of wet media milling and spray drying is presented in Table 1. Three different drug-stabilizer ratios, 10:1, 5.5:1, and 2:1 *w*/*w* (Factor A) and drug-mannitol ratios, 2:1, 1:1, and 0.5:1 *w*/*w* (Factor B) were tested. The obtained nanosuspensions were subsequently spray dried, employing inlet temperatures of 67 °C and 85 °C. A total of 18 runs were explored for single factor and interaction models to analyze the effect that the critical process parameters (drug-stabilizer ratio, drug-mannitol ratio, and SD inlet temperature), exert on the product material’s CQAs, namely the Z-average size and the Z-potential, as well as the redispesibility of solidified agglomerates.

Regarding the IBU Z-average particle size the standard least squares method of multiple linear regression was fitted to model the data. The effect of CPPs and the probability value (*p*-value) on the average particle diameter and on Z-potential were evaluated. ANOVA was implemented to assume a further cause–effect relationship; this infers that the factors sort the given data points into one of the groups causing the mean value difference. In addition, three-dimensional surface plots were constructed to visualize the CQA responses in relation to the two-factor interactions, while numerical optimization was employed to explore the design space in order to define the desirable operational space.

#### 2.2.9. Computational Simulation of the Solid State Properties

IBU was simulated with the intention to delve into the fracture mechanism, during milling. The energy frameworks defining the crystal structure of the APIs were simulated by a combination of computational chemistry techniques, which facilitate the understanding of intermolecular interactions that determine the mechanical properties of drugs.

##### Molecular and Solid State Modelling

The structural aspects and surface chemistry of IBU were investigated following a combination of approaches that provide insight into the intermolecular interactions that define the mechanical properties of the drug and surface adsorption of the stabilizer.

##### Crystal Morphology Modelling

Crystal morphology diagrams based on Bravais-Friedel-Donnay-Harker (BFDH) theory were constructed using the GDIS program [24], to facilitate the identification of crystal faces that are more likely to occur. The crystal faces identified as the most probable were further subjected to Attachment Energy calculations in order to construct the so-called growth morphology. Calculations were performed with the General Utility Lattice Program, GULP v.5.2 [25], using Dreiding 2.21 force field parameters [26] combined with high quality electrostatic potential-derived CHELPG atomic point charges calculated at the 6–31G**/MP2 level of theory by the Firefly quantum chemistry package (Alex A. Granovsky, Firefly version 8.2, http://classic.chem.msu.su/gran/firefly/index.html), which is partially based on the GAMESS US [27] source code.

##### Lattice Energy Frameworks

The intermolecular interactions in the crystal structure of IBU were analyzed using the semi-classical density sums (SDS-pixel) approach developed by Gavezzotti [28]. This method allows for a quantitative determination of lattice energy and pairwise intermolecular interactions, partitioning the energy into coulombic, polarization, dispersion and repulsion components. The interactions were further analyzed by constructing the so-called Energy Vector Diagrams [29]. More specifically, the interaction energy for each dimer within the first coordination sphere of the base molecule of IBU in the asymmetric unit (i.e., molecules with atom-atom distance shorter than the van der Waals radii sum plus 1 Å for at least one pair of atoms) was calculated as the difference between the energy of a dimer and the energy of the isolated monomers on the basis of Density Functional Theory calculations with the BLYP functional augmented by empirical dispersion correction (DFT-D) and def2-TZVP basis set, applying basis set superposition error correction with the Boys–Bernardi counterpoise procedure. Calculations were performed using the Orca quantum chemistry code [30] and the energy frameworks were drawn as energy vector diagrams (EVDs) or “hedgehogs” with the help of the CMOL collection of Python scripts for Energy Vector Diagram analysis of crystal structures [29]. Visualization of EVDs was effected using the Mercury software program [31].

##### Mechanical Properties

The energy of the crystal lattice of IBU was minimized with the GULP code [25] and the mechanical properties, namely the bulk (K) and shear (G) moduli, the Hill averages of the Young (E) moduli in three dimensions, and the compressibility, were calculated from the second derivative matrix. The universal elastic anisotropy index, A_u_, [32] was calculated according to the equation:

(3) $$A_{u}=5\frac{G^{V}}{G^{R}}+\frac{K^{V}}{K^{R}}-6$$

where R and V denote the calculation of the corresponding modulus following the Reuss or Voigt formula, respectively. The spatial dependence of the Young modulus, as well as of the compressibility, were visualized using the ELATE tool for analysis of elastic tensors [33], available online at http://progs.coudert.name/elate.

##### Surface Adsorption Simulations

The adsorption of Pharmacoat 603 (which is a low viscosity HPMC) and a molecule of IBU and mannitol were modelled for the (100) crystal face, which was found to be the most important morphologically, having a much larger surface area, following the simplified approach of Konkel and Myerson [34]. Each crystal face can be cut along more than a single plane parallel to the surface, giving rise to different surface terminations. The most stable face cut (the one having the lowest energy) was modeled in the present work. A 14 × 10 × 2 supercell was used and an oligomer of HPMC, consisting of 15 monomer units, was placed on top of a suitably sized surface slab, in a position close enough to allow for the development of intermolecular interaction with the surface, manifested by the formation of hydrogen bonds, the energy of the system was minimized, followed by a short molecular dynamics run at room temperature (298 K) for 150 ps. The process was repeated for a single molecule of mannitol and IBU instead of the HPMC 15-mer. While it has been suggested that monocarboxylic acids [35,36] crystallize as hydrogen-bonded cyclic dimers, polar solvents capable of accepting hydrogen bonds have been shown to disrupt the dimers [37], therefore a single molecule of IBU was chosen as the growth unit in this study, considering that the milling takes place in aqueous environment. All molecular dynamics (MD) simulations were performed within the NVT ensemble at 298 K by Langevin dynamics with periodic boundary conditions, using CHARMm force field parameters [38] in combination with Gasteiger partial atomic charges, and Particle Mesh Ewald electrostatics calculation. The dielectric constant of water (78.4) was used to simulate the effect of the aqueous environment on the electrostatic interactions. The molecular dynamics simulation setup allowed for the use of variable time step (1 fs for bonded, 2 fs for non-bonded, and 4 fs for electrostatic interactions). The modified impulse method (MOLLY) was employed to reduce the X-H bond resonance, in order to allow the application of time steps higher than 1 fs, according to the r-RESPA method [39]. All simulations were performed using the NAMD molecular dynamics code [40], operated through the VegaZZ graphical front end [41]. The molecular dynamics trajectories of the final part where the system reached equilibrium were sampled and the pair interaction energy (i.e., the energy of interaction between the additive and the surface) was calculated for the HPMC 15-mer, IBU, and mannitol molecule using the NAMD energy plugin of the VMD software [42].

##### Process Design

The DoE approach provides valuable insight into the relations between process parameters, formulation factors, and product CQAs by exploring the design space of operability. However, it presents limitations when facing the challenge of transitioning across scales, i.e., from the lab to industrial unit operations. Additionally, the FDA “cGMPs for the 21st Century” initiative under the framework directives ICH Q8–11, advocates the transition from descriptive approaches to first principle ones, based on the fundamental physics of processes. The term scalability refers herein to the prospect of increasing the system power or capacity by adding components in open loop mode. Digital twins can be used to study the scalability and transfer to industrial environment without expending large quantities of raw materials, energy, human and equipment resources. In our study, the modules of the Aspen Plus V9 software were chosen in order to build the digital simulation. The process modeling involved three parts: (1) process power and work estimation, (2) developing a model for the particle size distribution, and (3) modeling the spray drying process. For a thorough description see the Supplementary Materials.

## 3. Results and Discussion

As already mentioned, for the optimization of significant formulation factors and process parameters a DoE approach was applied. DoE is a systematic statistical approach of pharmaceutical formulation development, one that aims to elucidate product quality properties and process capability towards manufacturing efficiencies. This methodology performs sound quality risk assessment upon the experimental implementation of predefined empirical or calculated parameter settings. In this fashion, DoE guides robust formulation and manufacturing activities, facilitating root cause analysis by both identification and control of factors that influence the drug product quality response. Therefore, it reduces product variability while strengthening the industry’s ability to identify manufacturing hazards due to the scale up of operations.

Specifically, in the present study, the multi-level full factorial model was selected, investigating the effect of three levels for those independent variables whose effect is expected to be continuous, thus allowing for their optimization, while only two levels were considered sufficient for the spray drying temperature, since the effect of this factor is expected to exhibit a discontinuity defined by the melting temperature of ibuprofen. This type of DoE allows for the optimization of significant formulation factors, and the concomitant elucidation of the effect of a critical process parameter, while keeping the required number of experimental runs to a minimum.

### 3.1. Wet Media Milling

#### 3.1.1. Stabilizer Selection

**Figure 1:**
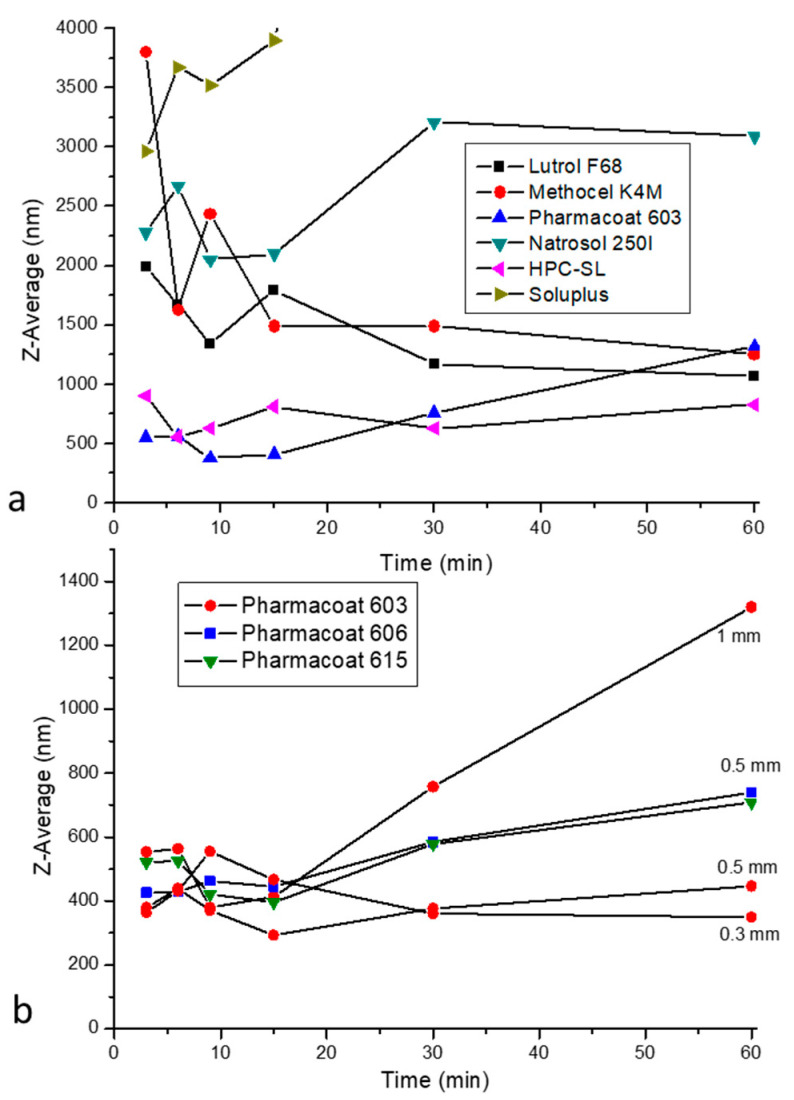
Time evolution of Z-average particle size of IBU: (**a**) in presence of different stabilizers, and (**b**) the effect of Pharmacoat solution viscosity (represented by different Pharmacoat grades), and bead diameter in presence of Pharmacoat 603.

The results of the mean hydrodynamic diameter, i.e., Z-average size over time, are shown in Figure 1. Evidently, the two most suitable stabilizers further investigated are the low viscosity cellulosic polymers, Pharmacoat 603 and HPC-SL. After an initial steep drop of the particle size at around 10 min of milling time, the Z-average size changes by a lower rate and after 60 min of processing it becomes 603 nm in the presence of Pharmacoat, and 828 nm in the presence of HPC-SL. Therefore, Pharmacoat 603 was selected as the stabilizing agent of choice. This finding is also in line with bibliographic data [43], suggesting that cellulose-type polymers are proven to be suitable for the stabilization of nano-suspensions. In the cases of Soluplus and PVP K12, size measurements by dynamic light scattering after 30 and 60 min did not produce credible results, probably due to extensive API dissolution.

#### 3.1.2. Effect of Stabilizer Solution Viscosity and Grinding Beads Diameter

Figure 1b illustrates the time evolution of IBU’s particle size in solutions of different viscosity with 0.5 mm beads, and the effect of different bead diameters in the presence of Pharmacoat 603. Regarding the effect of solution viscosity, Pharmacoat 603 also possessing the lowest solution viscosity, results in a more effective size reduction of IBU, compared to the other Pharmacoat grades. The effect of viscosity is not simple, as the higher viscosity grades, Pharmacoat 606 and 615 initially contributes to a more rapid particle size reduction, especially during the first 15 min.

Regarding the effect of bead diameter, it becomes evident that the decrease in diameter of the milling beads from 1.0 to 0.5 mm results in a significantly lower particle size of IBU nanoparticles. Further reduction of bead diameter does not seem to have a significant effect, while the handling difficulties in the sample after milling increases significantly. There was significant material loss noted during the final step of the collection of nanosuspension at the end of the milling process. Therefore, we decided to perform all subsequent milling runs with 0.5 mm diameter beads. For industrial scale up, this choice was later revisited.

#### 3.1.3. Correlation of Wettability and Surface Energy to the Particle Fracture

**Figure 2:**
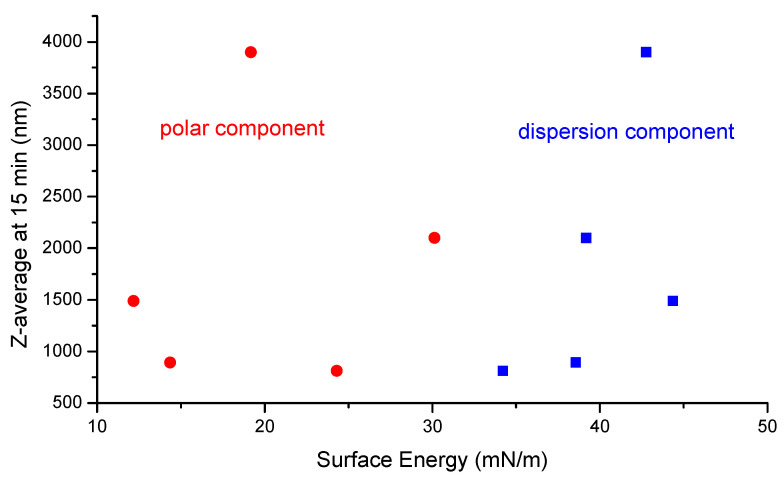
Correlation between Z-average size of IBU at 15 min of milling with the individual surface energy components of the stabilizers.

Contact angles formed between the surface of IBU and stabilizer solutions was measured at 60.2°, 55.7°, and 54.1° for HPC-SL, Pharmacoat 603, and Methocel K4M, respectively. Lower contact angle, and therefore improved wettability, corresponds to higher particle size and lower stabilization efficiency. This effect, however, is confounded with the effect of solution viscosity, since HPMC K4M solutions are more viscous than the corresponding solutions of HPC-SL and Pharmacoat 603 at the same concentration. Therefore, we considered this result to be inconclusive and attempted to correlate the stabilizer efficiency when reducing the particle size (referring to the Z-average size achieved after 15 min of milling), to the polar and dispersion surface energy components, as demonstrated by Table 2, while the results are exhibited by Figure 2. The dispersion component weighed contribution appears dominant at territories of high surface energy values.

#### 3.1.4. Selection of Diluent/Co-Milling Agent

**Figure 3:**
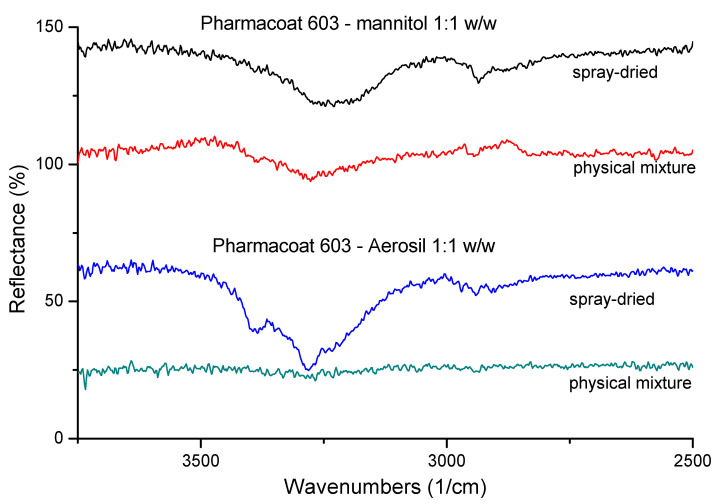
Representative ATR-FTIR spectra of 1:1 *w*/*w* physical mixtures of Pharmacoat 603 with mannitol and Aerosil before and after spray-drying.

ATR–FTIR spectra of dried mixtures of the selected Pharmacoat 603, with Mannitol and Aerosil, were employed to investigate possible intermolecular interactions (Figure 2). Mixtures with mannitol, independent of the concentration, showed significant differences before and after SD, and in particular a shift of peaks at 3350 and 3000 cm^−1^, attributed to vibrations of the C-H groups and of O-H bonds, respectively. On the contrary, mixtures with Aerosil produced spectra free of peak shifts or any other noticeable changes except for a peak intensity increase, which is probably a result of Mannitol recrystallization upon drying. These findings indicate the absence of molecular interactions induced by the SD process. Mannitol was selected as a suitable diluent, as it was deemed likelier to interact with Pharmacoat-603, due to the high reflectance levels illustrated in Figure 3.

### 3.2. Optimization of the Wet Media Milling Process

#### 3.2.1. Effects of Drug: Stabilizer Ratio on IBU’s Particle Size

The results of particle size for the WMM with Pharmacoat 603, as the stabilizer, are listed in Table 3 (numbering based on Table 1). The lowest particle size was obtained in Runs 2–13 and 4–12 where the ratio drug to stabilizer is set at 2:1. In Runs 5–11, 6–16, and 15–17 where the drug to stabilizer ratio is at the lowest analogy 10:1, the size could not be reduced below 1000 nm. In addition, there is no increase in particle size at any stage during the WMBM of IBU for all Runs, with the exception of Runs 10–18 post 30 min processing.

In Table 4, the ζ-potential of IBU nanocrystals is provided for each milling cycle. All runs demonstrate similar potential values, with slightly higher absolute values (and therefore improved stability properties) being observed for Runs 5–11, 2–13 and 10–18, where the content of mannitol is at its highest level, i.e., drug to excipient ratio of 0.5: 1.

#### 3.2.2. Solidification and Redispersion of IBU Nanosuspension

The SD process was implemented at two operating temperatures, 112% (85 °C) and 88% of the melting point (67 °C), according to the DoE (Table 1) and the dried material was tested for redispersibility, with results listed in Table 5. It should be noted that the DSC analysis of IBU samples coated with Pharmacoat 603 showed a clear melting point depression compared to the physical mixture (melting onset temperature 68.5 vs. 72.9 °C, respectively), further supporting the significance of the SD inlet temperature optimization. When dried below, the melting point of IBU, the nanosuspensions recovered their original Z-average size upon redispersion and the redispersibility index (RDI %) ranged from 80% to 105.2%. On the contrary, for inlet SD temperatures above the melting point threshold, agglomeration has a strong effect [44] increasing the particle size. When drying above the melting point, the redispersed nanocrystals showed increased size with RDI% ranging from 121.3% to 1088%. In some cases, and specifically for Runs 9, 15, and 11, it was not possible to measure particle size by dynamic light scattering because the observed values were outside the instrument’s operating range.

For inlet SD temperatures below the melting point threshold, the inner liquid escapes through the pores before it reaches the melting point in a rather complex dynamic fashion. The iterated droplet surface skin formation fosters solvent entrapment, and while shrinkage of an evaporating droplet takes place, dissolved and dispersed component concentrations increase at the surface. The concentration gradient causes a diffusional flux from the surface to the center of the droplet. Different sizes are obtained depending on which process, i.e., the transport to the surface or the diffusion towards the core [45] prevails.

Péclet (P_e_) is a dimensionless number that describes the diffusion time over a distance ratio to drying time (τ_dry_) given by

(4) $$P_{e}=\;\frac{\tau_{\mathrm{diff}}}{\tau_{\mathrm{dry}}}=\;\frac{\kappa }{8D_{i}}$$

κ being the evaporation rate and D_i_ being the diffusion coefficient of the Stokes–Einstein Equation [46]. The diffusion coefficient ratio corresponds to the reciprocal ratio of Péclet numbers if we assume equal evaporation rates. Plugging the diffusion coefficients of 6.6 × 10^−6^ cm^2^ s^−1^ for mannitol [47] and 9.8 × 10^−8^ for IBU nanoparticles, we get P_e,IBU_ = 67.3 × P_e,MN_ where P_e,IBU_ and P_e,MN_ are the Péclet numbers of IBU and mannitol, respectively. Consequently, mannitol is tending to diffuse towards the core during SD, while IBU motion is too slow to leave the droplet volume, thus limiting diluent and solvent entry to the droplet. Nevertheless, based on the above, it is reasonable to assume that mannitol still concentrates around the surface area, preferably where HPMC is absent, thus separating the formed nanoparticles and ameliorating their irreversible agglomeration [47]. In addition, the dissolved mannitol is crystallized at the surface of the droplet upon drying, i.e., prior to the critical point where electrostatic attractive nanoparticle forces overcome the repulsive ones [48], thus creating a matrix excipient barrier of particle movement by forming bridges between IBU particles, strongly enhancing the solid redispersion stability of the nanoparticles. Moreover, HPMC may act as a nucleation inhibitor for APIs possessing hydrogen-bond acceptor moieties, such as IBU (two acceptor groups) [49,50] and this effect is found to be concentration dependent [51]. This delaying nucleation feature is attributed not only to the required activation energy of nucleation increase, but also to the clear yet ambiguous reduction of crystal growth [52,53].

Regarding the ζ-potential, as demonstrated by Table 4, Runs 6–16 with a drug to stabilizer ratio of 10 and Runs 3–14 with a drug to stabilizer ratio of 5.5:1, showed a slight increase when drying was performed above the melting point. In all other cases the ζ-potential absolute value appears higher when drying at 67 °C. In relation to the starting material, the absolute ζ-potential values of all Runs are reduced after drying and therefore the stability of the mixture decreases. The effect of inlet SD temperature on the ζ-potential seems to depend on the drug to mannitol ratio. This is further supported by the fact that the ATR-FTIR spectra of each Run after drying above and below the melting point (spectra not shown) exhibited peak displacements at 3200 and 2900 cm^−1^ attributed to stretch vibrations of O-H bonds and C-H bonds, respectively, and in most cases, there is a reduction of peak intensity, which can be attributed to mannitol. The reduction of the intensity can be explained by the modification of the dipole moment upon recrystallizing above the melting point.

#### 3.2.3. Statistical Analysis of the Effects of Process Parameters and Formulation Factors on the CQAs of IBU

**Figure 4:**
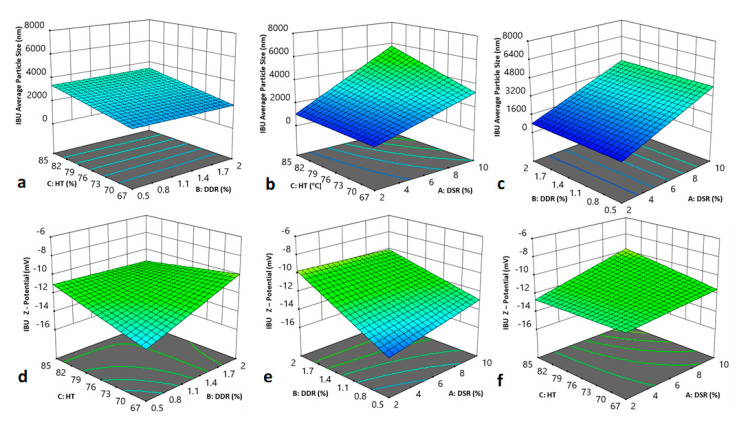
Response surface and contour plots of the main effects on the particle size vs: (**a**) inlet temperature and drug-mannitol ratio, (**b**) inlet temperature and drug-stabilizer ratio, (**c**) drug-diluent and drug-stabilizer ratio, and ζ-potential vs: (**d**) inlet temperature and drug-mannitol ratio, (**e**) drug-mannitol and drug-stabilizer ratio, and (**f**) inlet temperature and drug-stabilizer ratio.

The effects of the formulation factors and process parameters considered in the DoE, on the average particle size of the redispersed IBU suspensions, were statistically analyzed and their numeric results are presented in Table 6 and graphically by the three-dimensional orthogonal plots in Figure 4a–c.

The ANOVA study of statistically significant factors revealed that the increase of spray drying temperature significantly increases the particle size of the redispersed suspensions (*p*-value of 0.0493), Figure 4a. On the contrary, the drug to mannitol ratio did not significantly affect the size of the SD product, at least within the design space frame of the DoE. This result appears in line with the experimental findings (Table 5, Runs 2–13 and 4–12). Moreover, the promotional effect that the drug to stabilizer ratio (i.e., the reduction of HPMC concentration) poses on the particle size of the dried IBU, bearing a p-value of 0.054, becomes apparent in Figure 4b. This effect is in line with our experimental findings (Table 5, Runs 2–10 and 4–5). An increase of inlet SD temperature also results in increased particle diameter.

**Figure 5:**
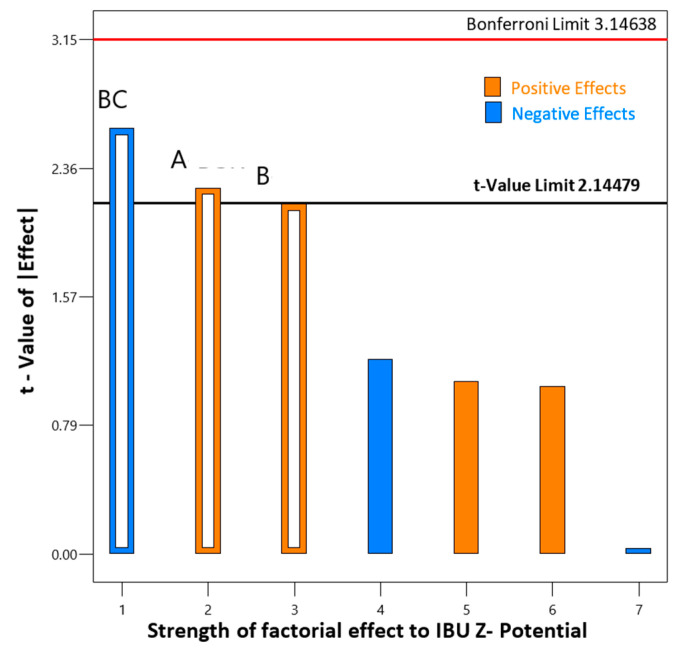
Concave frequency of occurrence of factor main and/or interaction effect versus the cumulative percentage of the total number of occurrences; the two factor interaction BC, and main effects of factors A and B are deemed significant.

Regarding the effects on the ζ-potential, the ANOVA results displayed in Table 7, reveal an obtained F—value of 3.47, which suggests that the proposed model is significant, i.e., there exists a 3.56% chance that the F-value would occur due to noise, when p-values of less than 0.05 are deemed significant. Since probable two-factor interactions are also analyzed, a Pareto test chart was initially graphed to quantify the factorial significance, presented in Figure 5. The source of variance was further calculated for the two-factor interaction model, i.e., the factors alone, the interaction between them, residual, lack of fit, pure error and correction total. The interplay between inlet temperature and drug-mannitol ratio, bearing a p-level of 0.0290, ascends as the dominant combined effect on the particle ζ-potential.

Expanding on this, as demonstrated by the response surface plot of Figure 4d, the increase of mannitol concentration, coupled with a temperature drop below that of the applied inlet temperature, defines the effects of the absolute ζ-potential increase, hence stabilizing the nanocrystals. In addition, drug-mannitol and drug-stabilizer ratios, presenting p-values of 0.0587 and 0.0570, respectively, appear to contribute almost equally, as shown by the plot of Figure 4e, to the ζ-potential variance, elaborating that the increase of either HPMC or mannitol concentration enhances the nanosuspension stability, and contributes to lower particle size and to a higher dissolution rate. The predicted linear relationship for the ζ-potential was assessed and the coefficient estimates, which represent the expected variance in response per unit change in the factor value when all remaining factors are held constant, are shown in Table 8.

#### 3.2.4. Design Space Optimization for the Lab Scale Production of IBU Nanocrystals

**Figure 6:**
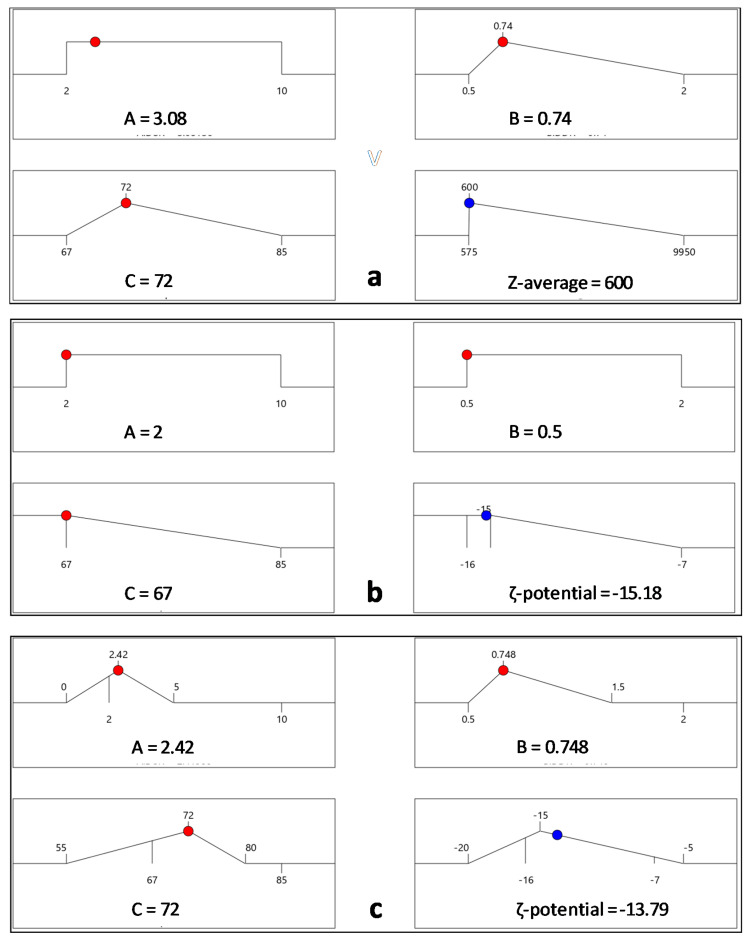
Numerical optimization ramps showing the optimal solution that meets the criteria of desired CQAs. The optimal factor settings of inlet temperature, drug-stabilizer and drug-mannitol ratios are shown with red dots, while the optimal response prediction values are displayed in blue: (**a**) Average particle size set minimum at 600 nm; (**b**) algebraic corresponding ζ-potential at −15,187 mV and set minimum inlet temperature corresponding to 67 °C; and (**c**) the combined optimal response prediction of A and B according to the QbD iterated standards.

The CQA optimization criteria for the responses Z-Average size and the ζ-potential taking into account their third order interactions with variables of the drug-stabilizer ratio (Factor A), drug-mannitol ratio (Factor B) and inlet temperature (Factor C), are represented by the destination ramps of Figure 6a–c.

**Figure 7:**
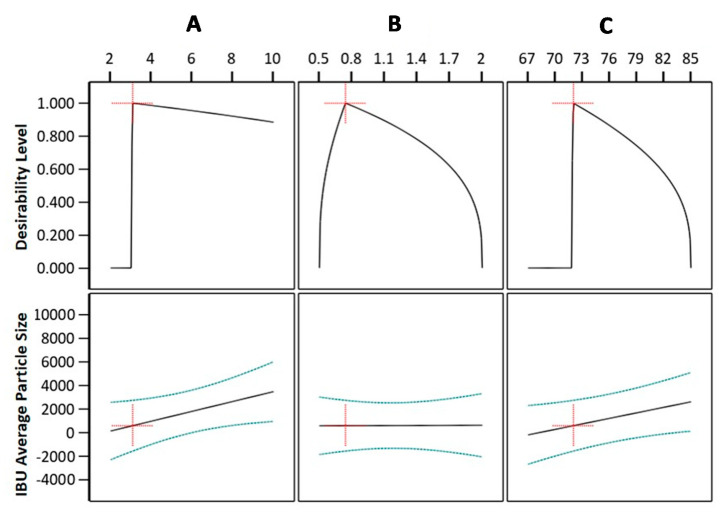
Desirability graphs of factors (**A**), (**B**) and (**C**), plotted against the IBU average particle size. The red crosses of the first row graphs correlate the achieved a desirability value of the y-axis against the *x*-axis predicted parameter value. The red crosses of the second row graphs correlate the first row values of desirability and each factor to the IBU average particle size CQA of 600 nm.

Regarding the Z-average size, a suggested solution of desirability value is demonstrated in Figure 7 against Factors A, B and C.

**Figure 8:**
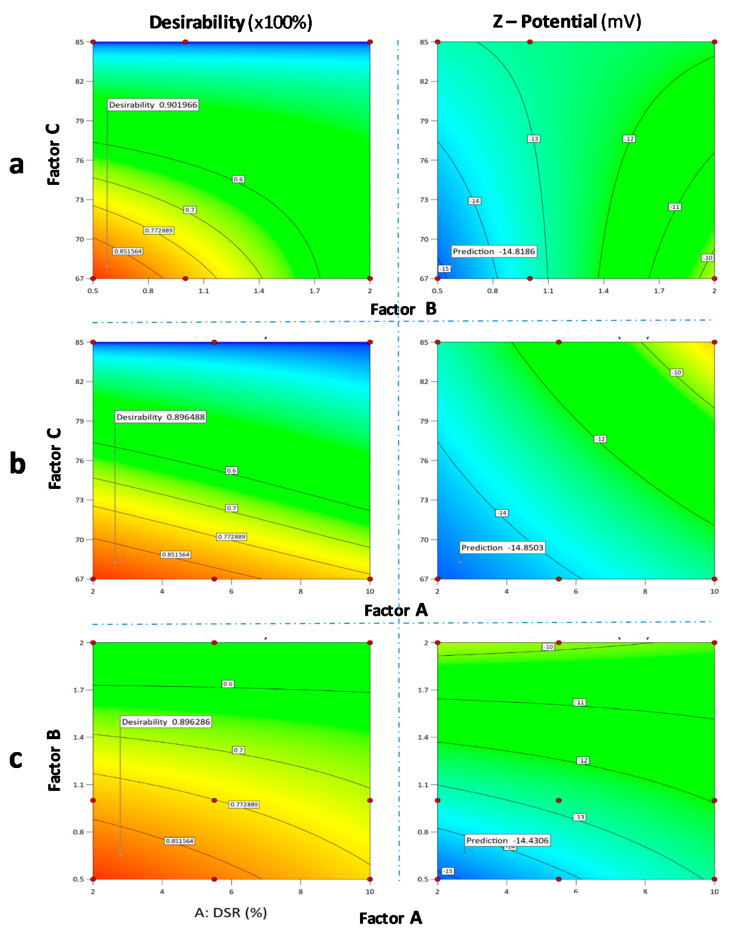
Contour plots of the desirability function used to explore the factor interaction space of: (**a**) inlet temperature and drug-mannitol ratio, (**b**) inlet temperature and drug-stabilizer ratio, and (**c**) drug-stabilizer ratio and drug-mannitol ratio. For each graph on the left column, one random yet correlated to the optimum individual ζ-potential response, is flagged. Red to orange contours converge to the operational area of interest. For each graph on the right column, the optimum respective individual ζ-potential response is flagged. Shades of blue contours converge to the operational area of interest of the selected displayed factors.

Regarding the ζ-potential, a suggested solution of desirability projected value of 0.954 is demonstrated in Figure 8a–c when the absolute desirability is given as a value approximate to 1.00 and is plotted against the inlet temperature and drug-mannitol ratio, inlet temperature and drug-stabilizer ratio, and drug-stabilizer ratio and drug-mannitol ratio, respectively. These models, created by the combination of the calculated desirability levels for the Z-average size and ζ-potential, are then overlaid to scan the design space.

**Figure 9:**
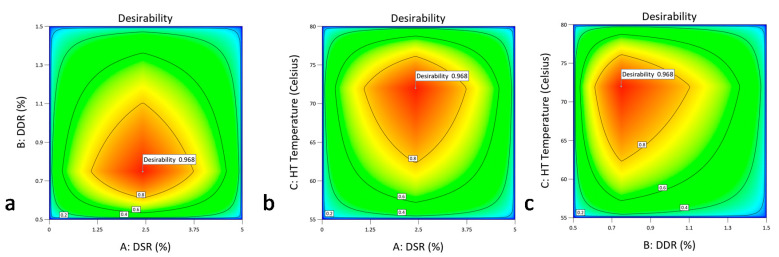
Contour plots of desirability function used to explore the overall factor interaction space for the combinatory fullfilment of all the CQA criteria: (**a**) drug-stabilizer ratio and drug-mannitol ratio, (**b**) inlet temperature and drug-stabilizer ratio, and (**c**) inlet temperature and drug-mannitol ratio.

Having statistically defined the DoE CPP effects and the QbD operational space of the controlled variables that meet the CQA criteria of Figure 6 for both the main unit operations and combinatory overall suggested solution of excellent desirability projected value of 0.968 for DSR and DDR, HT and DSR, HT and DDR, is displayed in Figure 9a–c, thus allowing for the configuration of the overall optimum material specifications and temperature SD conditions. Our design space optimization showed that the IBU nanocrystals of approximately 560 to 600 nm average hydrodynamic diameter and a ζ-potential between −14.4 to −15,876 mV, are most efficiently obtained in the lab scale employing IBU to HPMC and IBU to mannitol optimum ratio ranges between 2 and 2.415% and 0.5 and 0.749% at preferred temperatures spanning from 72 to 73 °C.

### 3.3. Computational Investigation of IBU Fracture Mechanism

#### 3.3.1. Crystal Morphology

**Figure 10:**
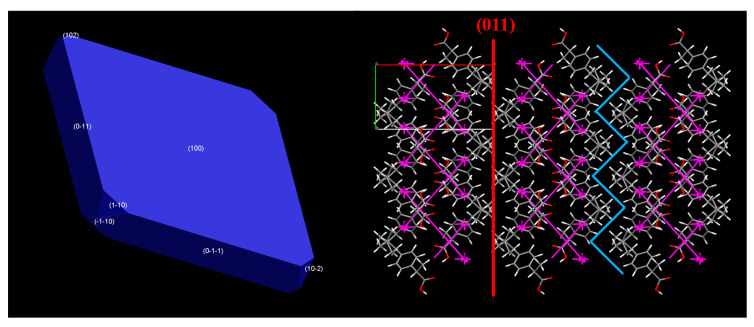
Growth morphology (attachment energy-based) of IBU crystals and energy vector diagrams (EVDs) depicting the total interactions in the unit cell, viewed along the c-axis.

Figure 10 illustrates the attachment energy (growth) morphology model of IBU crystals, together with EVD plots viewed along the c-axis of the unit cell. The predicted morphology is in good agreement with previously published attachment energy morphologies [54,55], and emphasizes the morphological importance of the (100) crystal face. On the basis of the growth morphology model, the (100) and (0–11) faces were chosen for the stabilizer adsorption study, as they are the only ones with sufficient morphological importance (sufficient surface area) to act as adsorption sites for any additive.

#### 3.3.2. Lattice Energy Frameworks

SDS-pixel energy calculations partition the total energy of −1111.9 kJ/mol to a strong polarization component (−1114.3 kJ/mol), followed by the dispersion (−90.3 kJ/mol), and Coulombic (−24.6 kJ/mol), with a weak repulsive component (117.2 kJ/mol). This can be visualized in the EVD plots, Figure 9. EVD plots show that the strongest interactions are formed between the hydrogen bonded dimers of IBU. When viewed along the c-axis of the unit cell, they further indicate the existence of a possible slip direction along the (100) Miller plane (red line). However, the topology of the lattice suggests that substantial steric hindrance is expected to slip along this direction, because of the high rugosity of the molecular layers forming the plane (blue line). An additional reason limiting the effectiveness of the (011) slip plane can be the small surface area of the corresponding crystal facet, as this is expected to greatly reduce the probability of impact of a grinding bead on the specific crystal facet, which would cause a fracture along the (011) slip plane.

#### 3.3.3. Mechanical Properties

**Figure 11:**
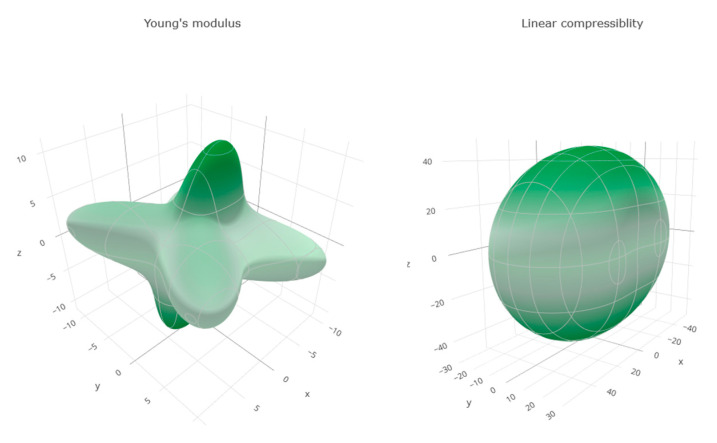
Vector plots illustrating the spatial dependence of Young’s modulus and linear compressibility.

Table 9 lists the calculated mechanical properties, and Figure 11 illustrates the spatial distribution of Young modulus and compressibility index tensors. From the results of Table 9, it is seen that the anisotropy index is rather low, indicating the absence of preferred slip directions. The values of the elastic constants suggest a rather soft lattice with a high compressibility, in accordance with the known ductility of IBU. Therefore, plastic deformation rather than brittle fracture seems to be more likely when the lattice is strained, which can explain, at least partially, the difficulty in comminuting IBU crystals down to the nanoscale.

#### 3.3.4. Surface Adsorption Simulations

**Figure 12:**
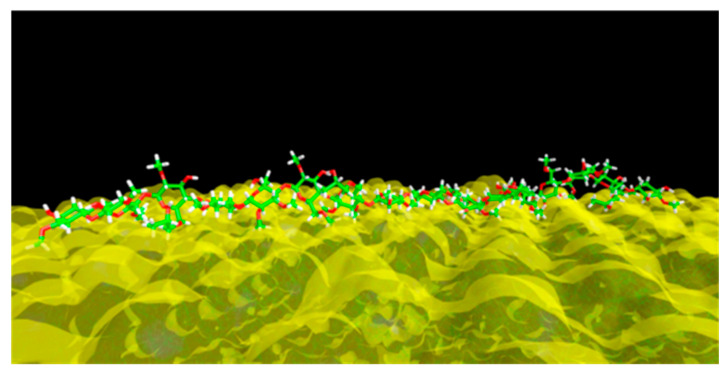
Adsorbed HPMC molecule on the (100) face mapped with an isosurface colored according to the molecular electrostatic potential (MEP).

A snapshot of the trajectory of the MD simulation is illustrated in Figure 12, showing that the HPMC molecules interact with the (100) crystal face mainly via the hydrophobic methyl groups by dispersion interactions, while the hydroxyl moieties are oriented towards the aqueous phase. The pair interaction energy between surface and stabilizer (E_bind-st_) was found to be −133.4 kcal/mol, while the corresponding interaction energies between the surface and drug (E_bind-dr_) and surface and mannitol (E_bind-mann_) were found −13.2 kcal/mol and −9.2 kcal/mol, respectively. This result indicates that HPMC has roughly a tenfold higher affinity to the (100) surface than IBU molecules, while mannitol competes with IBU with a nearly equal binding affinity. These results suggest that HPMC acts as a potent crystal growth inhibitor, minimizing the Ostwald ripening effect, while a steric stabilization is also expected due to the coverage of the particles’ surface and prevention of strong agglomeration between the nanocrystals [56]. However, at least in the timeframe of this simulation and with the selected force field and statistical ensemble, no local disorder or other evidence of disruption of the crystal surface’s intermolecular interaction framework was observed that could be interpreted as an indication of an adsorption-induced reduction of strength (AIRS or Rehbinder effect). Therefore, it can be inferred that HPMC acts mainly as a steric stabilizer, as well as an inhibitor of Ostwald ripening.

### 3.4. Process Design

#### 3.4.1. Modelling of the Wet Media Milling Process

**Figure 13:**
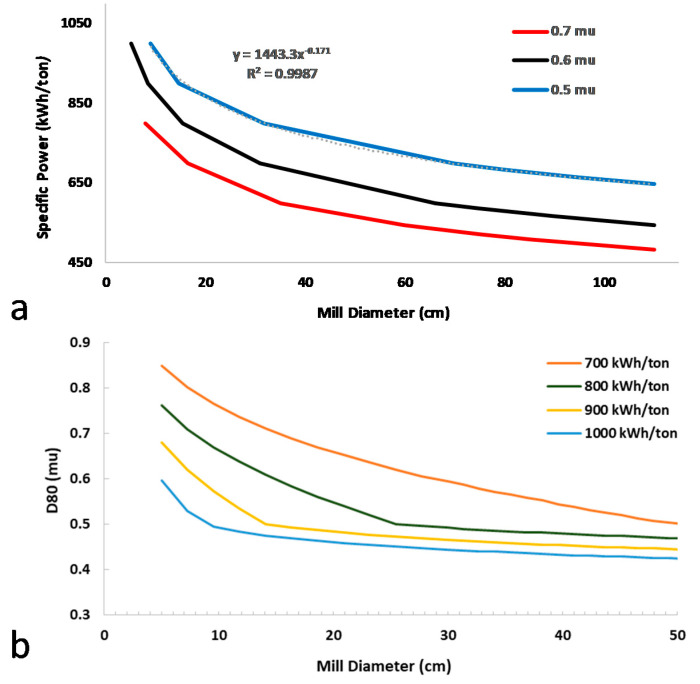
(**a**) Theoretical dependence of the specific power on the diameter dimension of the ball mill, and (**b**) correlation between the *d80* and the mill diameter for different specific power inputs.

In order to explore the scalability of the formulation approach described so far, an algorithm was designed in Aspen Plus V9 simulator, which allows for the estimation of the critical process parameters and the sizing of a ball mill in the context of a scale up process capable of producing 12 tons/year. The variables studied were categorized as: (i) flexible, namely mass throughput, d80 diameter, mill diameter, and specific power; (ii) fixed namely product filling ratio, grinding ball filling ratio, critical and angular speed relationship, length to diameter ratio, BWI; (iii) calculated, namely angular speed, power, mill length, residence time, and mass of grinding balls. In agreement with the experimental data and desirability of the CQA criteria, the ball mill was assumed to be operating continuously for the process time of one hour to deliver particles with d80 of 600 nanometers. The d50 particle diameter of micronized IBU inlet starting material was considered to be 100 μm, assuming normal distribution with *X* standard deviation of 10 μm, and a Bond Working Index, BWI, of 8 kWh/ton, according to Aspen V9 data bank. Electrical to mechanical efficiency was set at 80% according to previous experimental results reported [57]. Plots of the theoretical dependence of the specific power on the diameter dimension of the ball mill, and the relevance between the mill diameter and the d80, for a variety of a specific power inputs, in order to meet the given CQAs, are given in Figure 13a,b, respectively.

The diagrams tailored to the QbD WMBM process demonstrate the optimum operating area of the planetary mill, based on Rittinger’s Law and the Rosin-Rammler distribution. Beginning with the specification of the characteristic size of the IBU product, an advantageous size range of the ball mill is distinguished. By increasing the milling jar diameter above a certain value, the input of specific power becomes irrelevant, while decreasing the diameter of the ball mill below a certain value; there is no requirement of a higher specific power in order to achieve the same result. The working bowl volume was designed to fill three times the volumetric space of material throughput [58], while the height of the jar (h) in respect to the diameter (d) was calculated as

h = 0.6449 × D,(5)

so that

(6) $$d=\sqrt[{3}]{\frac{4\times V}{\pi \;\times 0.645}}$$

The mill bowl diameter was initially calculated at 0.6 m, but due to the above design observations and hardware limitations, a d of 0.2118 m was deemed to be preferable. This choice increased the required estimated number of ball mills to 10 and the number of bowls installed per each mill to 4.

For the desirable IBU product of *d80,* a diameter of 600 nm was the correlated specific power was calculated by the simulator at 2700 KJ/kg. The mill bowl height (h) was calculated to be 0.1365 m, albeit the ratio used was d/h = 0.6449, jar volume (*V*) to 4812.38 cm^3^, weight of feed (m) to 0.2127 Kg, sun disk rotation speed (Ns) to 240 rpm, bowl rotation speed (Nb) to 250 rpm and radius of sun disk (*R*) to 0.3 m. Due to the heat generation, only 60% of the given power is assumed to actually be transferred to the solids and the milling beads. The critical ball mill speed, i.e., the theoretical rotational speed at which the centrifugal force on a ball in contact with the mill shell at the height of its path equals the force of it due to gravity being calculated. The critical speed was given as a function of the diameter (*ω_c_* = 42.3 × D^0.5^). The angular speed fueled by the Coriolis forces was considered to be 80% of the critical speed [57]. The spherical milling beads were set to the commercial existing dimension of d = 0.008 m and the loading level was adjusted to fill the 1/3 of the working pot volume [57]. The ball-size feed distribution was calculated as a rationed ball charge [59] according to

(7) $$D_{b}=\;\sqrt{\frac{X_{\mu \;}{Ε}_{i}}{143n_{r}}}\;\sqrt{\frac{\rho_{s}}{\sqrt{d}}}\;,$$

where $D_{b}$ is the ball diameter in cm; d is again the mill diameter, m;${\;Ε}_{i}$ is the BWI; $n_{r}$ is speed percent of critical; and $\rho_{s}$ is the feed specific gravity. The process parameters critical for a scale up, i.e., the product dependent BWI and specific power, the mill diameter, which defines the correction of the BWI, the mass throughput, the angular speed defined by the critical speed of the mill, and finally the type and quantity of the grinding balls dependent of the rest of the parameters are provided in Table 10.

#### 3.4.2. Modelling of the Spray Drying Process

**Figure 14:**
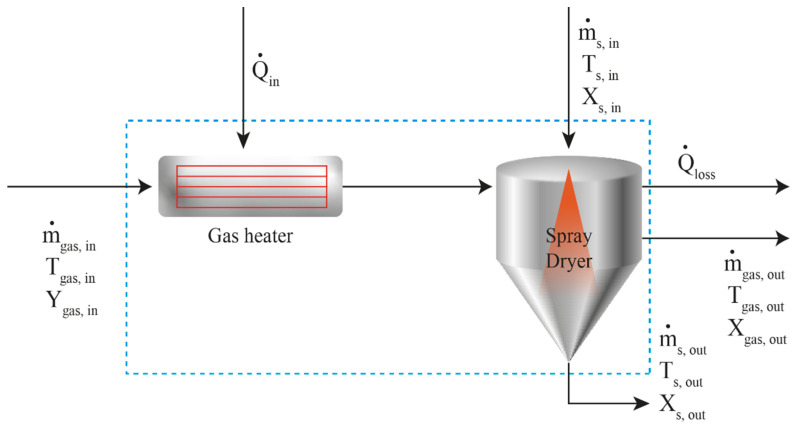
Schematic representation of mass, energy and enthalpy balances taking place in SD process: the drying medium flowing in at rate $\dot{m}$_gas,in_ is pre heated at duty $\dot{Q}$_in_. The solids and liquid mixture enter the spray tower at rate $\dot{m}$_s,in_. The outlet material flows at rate $\dot{m}$_gas,out_ and $\dot{m}$_s,out_. The amount of $\dot{Q}$_loss_ is expended to the environment. The overall mass balance is calculated by the equation $\dot{m}$_gas,in_ + $\dot{m}$_s,in_ = $\dot{m}$_gas,out_ + $\dot{m}$_s,out_.

The equations of the overall mass balance, moisture and enthalpy of solid/liquid IBU mixture were calculated under the reiterated scheme, as shown in Figure 14, for a total residence time of 1 min in agreement with our experimental data. The volumetric air inlet (cum/h) divided by 60 min produces the ratio of gas volume per minute, which is considered equal to the volume of the SD chamber. The optimum design length to the diameter ratio is 4, while the nozzle angle suitable for thermosensitive material is preferably 60 degrees [60], so in relation to the design, the equation takes the following form:

(8) $$V_{\mathrm{chamber}}=\;\frac{\pi }{4}{\;D}^{2}\left(H+\frac{\sqrt{3}}{2}\;D\right)$$

and for H = 4 × D then V = 3.82 × D^3^ and consequently $D=\sqrt[{3}]{\frac{V}{3.82}}$ resulting in a proposed SD diameter *D* of 0.93 m and a height *H* of 3.72 m. At these dimensions, two spray dryer units are needed to match the production goal of 12 tones/year due to nozzle and atomizer space requirements.

**Figure 15:**
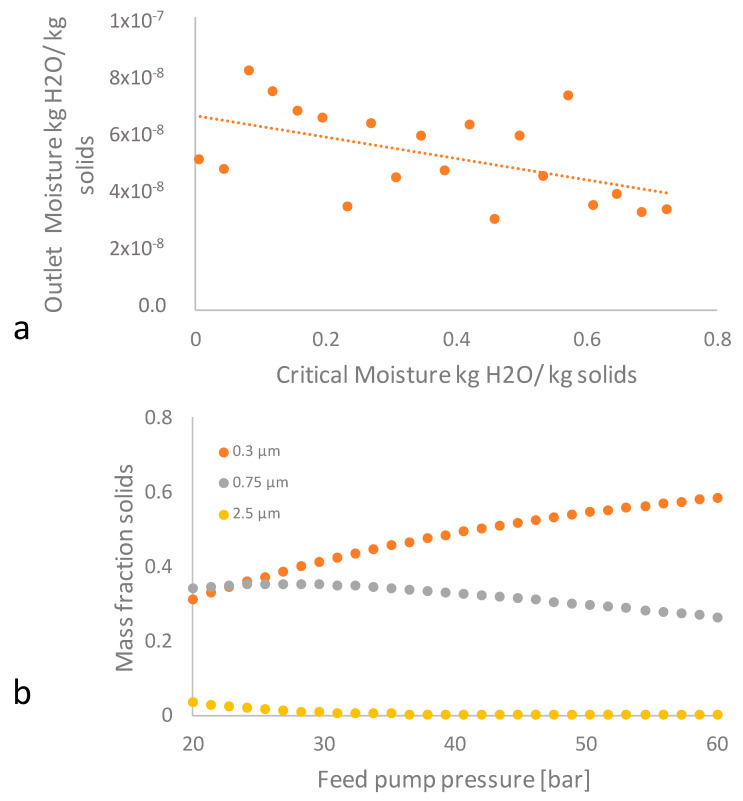
Sensitivity analysis at constant temperature of 52 °C: (**a**) of dependence of critical moisture to outlet product IBU moisture content demonstrating SD efficiency, and (**b**) of feed pump pressure dependence on the mass fraction IBU solid.

Figure 15a illustrates the moisture content of IBU material, extracted by Aspen sensitivity analysis, keeping the temperature constant to 52.3 °C, expressed in absolute terms of kg moisture/kg dry substance IBU, i.e., equal increments in percentage moisture represent equal changes in weight. For critical moisture 0.044 kg H_2_O/kg solid, an initial inlet moisture content is 69.6 kg H_2_O/kg solid and so the inlet moisture IBU content is measured three orders above the critical value, thus ensuring that the whole process is outside the falling-rate zone. During the SD process, the solid/liquid mixture is droplet-sprayed, interfacing the warm vapor that removes the liquid by evaporation. In the beginning, the droplet’s temperature increases, through the convection sine, the evaporation contribution. Once the temperature reaches the wet bulb threshold estimated by Aspen V9.0 at 53 °C, the liquid phase starts evaporating while the droplet temperature changes moderately. Close to the critical content moisture point of 69.6 Kg H_2_O/Kg a crust of IBU and of excess HPMC is formed and the droplet transforms to a moist particle, hence evaporation is hindered by mass transfer delay through the pores of the crust, due to particles’ surface coverage. This coating is responsible for the prevention of agglomeration, which is then translated to the size reduction, when the concentration of HPMC increases in the mixture. The latter assumption is in line with our experimental findings (Runs 2–10 and 4–5).

Moreover, the predicted outlet moisture ratio is 6.09 E-8 kg H_2_O/kg, fulfilling the QbD set conditions for the minimum water content. Figure 15b illustrates the sensitivity analysis of feed pump pressure dependence on the mass fraction IBU solid. It is seen that the mass fraction of solid IBU undergoes an increase in the 0.3 μm PSD15 mass fraction and a simultaneous decrease of the 0.75 μm mass fraction, translated to a desirable diminution of particle size when the pressure is raised. Operational pressure of a centrifugal pump model was then set to 45 bar in order to meet a 0.517 mass fraction IBU solid of 0.3 μm PSD15 in line with QbD.

**Figure 16:**
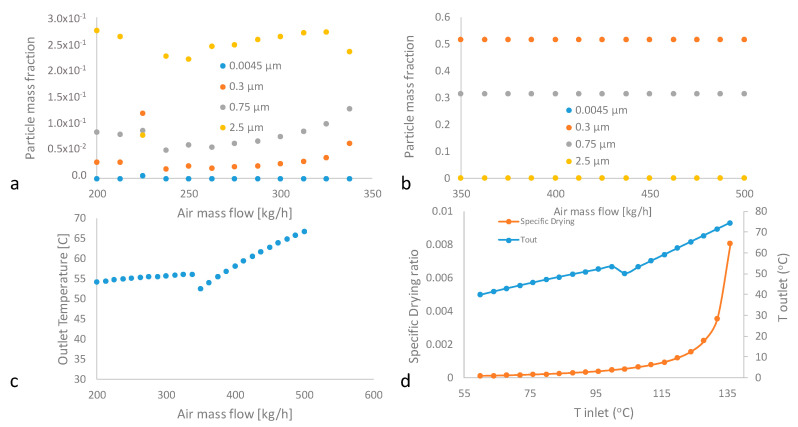
Sensitivity analysis of: (**a**,**b**) particle mass fraction dependence on different ranges of air mass flow, (**c**) outlet temperature dependence on air mass flow and (**d**) specific drying ratio dependence on inlet temperature.

In Figure 16a,b, the sensitivity analysis of IBU particle mass fraction dependence on air mass flow when the SD temperature fluctuates between roughly 50 and 70 °C, is presented. The plot reveals that the SD air mass flow of less than 337.5 kg/h results in unstable, and moreover, incomplete drying operation with the moisture IBU ratio ranging from 52.703 to 1499 kg H_2_O/kg dry. When the critical mass flow threshold of 350 kg/h is reached, the SD process exhibits stability, combined with a favorable moisture ratio 7.19 × 10^−8^ Kg H_2_O/kg dry and in addition to the IBU product particle distribution approaches to 0.5 PSD15 of 0.3 mu and 0.32 PSD16 of 0.75 mu.

In Figure 16c, the effect of SD drying gas inlet air temperature on solid to air moisture ratio is provided. Solid to air moisture attains its minimum value at the inlet air temperature of 104 °C. The air inlet operational space between 104 and 130 °C was therefore investigated for the operating optimum. In Figure 16d, the correlation between air inlet and outlet SD temperature is provided. In Table 11, the air inlet and outlet temperature, IBU and gas moisture content, air and solid moisture feed and solid to air moisture are presented. According to our set QbD desirability criteria, demonstrated in Figure 7, the operational space now becomes evident.

#### 3.4.3. Process Flow Diagram for the Scale-Up of IBU Production for 12 Tons/Year

**Figure 17:**
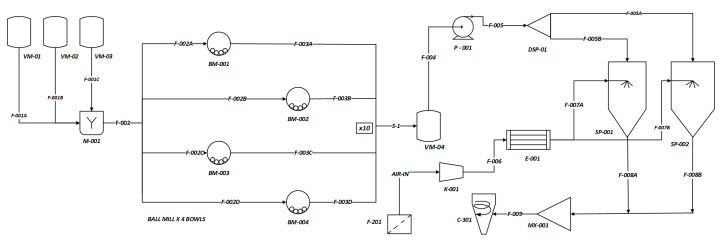
Process flow diagram nomenclature; *VM-01*: IBU vessel, *VM-02*: Mannitol vessel, *VM-03*: Pharmacoat 603 vessel, *VM-04*: Vessel container; *BM-001, BM-002, BM-003, BM-004*: Mill bowl; DSP-01: F-SPLIT; P-001: Pump; F-201: Air Filter; K-001: Compressor; H-001: Heater unit; SP-001, SP-002 Spray Dryer Unit; M-001: Mixer, MX-001: Flow chamber, C-301: Cyclone.

Having completed the DoE, the QbD study and modelling of the ball mill and spray drying, the final process flow diagram according to ISO 10628-1 that connects all the process unit blocks and the auxiliaries is presented in Figure 17. The manufacturing capacity of IBU nanocrystals is predicted to cover a pilot production of 12 tons of formulated API, in 301 days operating at a single 8 h shift, suggesting that further scale-up of IBU nanocrystal is feasible. Each ball mill complex process unit is represented by the rectangular stripped shape that incorporates the BM-001 to BM-004 bowls, and hence is multiplied by 10 to meet the required production capacity.

## 4. Conclusions

The first multi-faceted study, which is carried out correlating computer aided mechanical and thermal process design with molecular modelling and experimental validation of IBU nanocrystal formulations is presented here. Moreover, this is the first study that investigates spray drying at temperature operations approaching the API melting point, in order to efficiently improve IBU’s solubility properties. Our experiments showed that IBU nanocrystals of approximately 560 to 600 nm average hydrodynamic diameter and a ζ-potential between −14.4 to −15,876 mV, can be efficiently obtained in a lab scale employing IBU to HPMC and IBU to mannitol optimum ratio ranges between 2.415 to 2% and 0.749 to 0.5%, respectively, after 60 min of wet media milling at 400 rpm with 70 g of 0.5 mm diameter beads, coupled with SD processing with an air flow rate of 800 m^3^/h, aspirator 80%, pump speed 5% and inlet air temperature set between 67–72 °C. Our results were then transferred to our computer aided process model design, which predicted the feasibility of actual manufacturing of the IBU nanocrystals at 12 tons/year capacity, when operating 10 ball mills incorporating four mill bowls, each of (d) 0.2118 m, (h) 0.1365 m, (V) 4812.38 cm^3^, at 0.2127 kg (m), (Ns) 240 rpm, (Nb) 250 rpm, loaded by 5.385 zirconium beads of 0.008 m diameter each and coupled with two industrial custom spray dryer units that were 0.930 m in diameter and 3.722 m in height, and remain connected to a centrifugal air pump, set to 45 bar, forcing a mass air flow of 350 kg/h, preheated to approximately 104 °C.

Several limitations apply to our study. Due to material loss issues, the experimental design continued with 0.5 mm diameter beads. The scale-up of unit operations is not a trivial task, hence KPPs affect the process and each other simultaneously. The algorithm of wet media milling process simulation may not work for a high residence time, hence in that case, the product claims most of the specified power, leaving less available grinding ball space. We tried to compensate for the same capacity using in parallel more than one ball mill, in the model. Also, we did not have the chance to validate the scalability assumptions of the process design with actual industrial results.

However, a substantial amount of novel APIs deal with inadequate solubility issues, of which thermo-sensitive and eutectic substances pose unsolved formulation and cost-related scale-up challenges. The combinatory methods presented herein may be further applied to cover DoE to first principle formulation and scale-up studies of such ductile, BCS class II drugs, featuring thermosensitivity and eutectic tendency.

## Figures and Tables

**Table 1:** DoE for the WMM and SD of IBU, using Pharmacoat 603 as stabilizer and mannitol as a co-milling agent.

Std	Run	Factor A IBU/HPMC (*w*/*w*)	Factor B IBU/Mannitol (*w*/*w*)	Factor C Inlet Temperature (°C)
8	1	5.5	2	0.88 × T_m_
1	2	2	0.5	0.88 × T_m_
5	3	5.5	1	0.88 × T_m_
4	4	2	1	0.88 × T_m_
3	5	10	0.5	0.88 × T_m_
18	6	10	2	1.12 × T_m_
7	7	2	2	0.88 × T_m_
16	8	2	2	1.12 × T_m_
17	9	5.5	2	1.12 × T_m_
2	10	5.5	0.5	0.88 × T_m_
12	11	10	0.5	1.12 × T_m_
13	12	2	1	1.12 × T_m_
10	13	2	0.5	1.12 × T_m_
14	14	5.5	1	1.12 × T_m_
15	15	10	1	1.12 × T_m_
9	16	10	2	0.88 × T_m_
6	17	10	1	0.88 × T_m_
11	18	5.5	0.5	1.12 × T_m_

**Table 2:** Polar, γ^p^_sv_, and dispersion, γ^d^_sv_, components, together with the total surface free energy, γ_sv_, (in mN/m) of stabilizers determined by the contact angle method.

Stabilizer	γ^p^_sv_	γ^d^_sv_	γ_sv_
Natrosol	30.14	39.19	69.33
HPC-SL	24.30	34.22	58.52
Pharmacoat 603	14.36	38.56	52.92
HPMC K4M	12.18	44.36	56.54
Soluplus	19.17	42.77	53.70
Ibuprofen	36.60	24.40	51.00

**Table 3:** Z-Average diameter of IBU during milling with Pharmacoat 603, according to the experimental design.

Z-Average (nm)/Run Number									
Time (min)	2–13	4–12	8–7	10–18	3–14	1–9	5–11	17–15	6–16
3	1620	3690	1910	4100	6530	6970	4140	6890	7120
6	1090	1120	1330	1950	3270	2500	2770	2820	3630
9	1350	712	1290	1860	2700	1990	3150	2230	2470
15	1010	680	1190	977	2280	880	2490	1570	2620
30	755	608	979	1120	1200	790	1620	2000	2570
45	752	438	909	832	714	1020	2070	1680	1570
60	462	444	750	648	741	837	1750	1320	1090

**Table 4:** ζ-potential of IBU nanocrystals in presence of Pharmacoat 603, after 60 min of milling.

Run Number	Ζ-Potential (mV)
3–14	−24.5
5–11	−34.0
9–1	−27.6
10–18	−27.5
17–15	−27.6
6–16	−27.3
4–12	−23.2
7–8	−24.6
2–13	−28.3

**Table 5:** Particle size, redispersion index, and ζ-potential of IBU before and after drying.

Spray Dried at 0.88 T_m_ (67 °C)				Spray Dried at 1.12 T_m_ (85 °C)			
RUN	Z-Average (nm)	RDI (%)	ζ-potential (mV)	RUN	Z-Average (nm)	RDI (%)	ζ-Potential (mV)
1	987	117.9	−11.7	9	9110 *	1088	−11.3
16	1200	110.1	−9.38	6	1400	128.4	−10.3
17	1360	103.0	−14.3	15	9950 *	753.8	−11.4
3	704	95.0	−12.1	14	899	121.3	−13.8
5	1960	112.0	−11.6	11	8860 *	506.3	−7.95
7	727	96.93	−12.5	8	940	125.3	−9.16
10	691	106.6	−16.0	18	1010	155.9	−12.5
2	575	124.5	−14.4	13	904	195.7	−12.6
4	642	144.6	−14.9	12	950	214.0	−13.8

* Size values were determined by optical microscopy.

**Table 6:** Analysis of variance for the statistical significance of the investigated factors (A: drug to stabilizer ratio, B: drug to mannitol ratio, C: inlet temperature) on the particle diameter after redispersion of the suspensions.

Source	Sum of Squares	df	Mean Square	F-Value	*p*-Value
Model	6.87 × 10^7^	3	2.29 × 10^7^	3.01	0.0656
A	3.35 × 10^7^	1	3.35 × 10^7^	4.41	0.0544
B	6862	1	6862	0.0009	0.9765
C	3.52 × 10^7^	1	3.52 × 10^7^	4.63	0.0493
Residual	1.06 × 10^8^	14	7.60 × 10^6^		
Total	1.75 × 10^8^	17			

**Table 7:** Analysis of variance for the statistical significance of the investigated factors (A: drug—stabilizer ratio, B: drug—mannitol ratio, C: inlet temperature) on the ζ-potential after redispersion of the suspensions.

Source	Sum of Squares	df	Mean Square	F-Value	*p*-Value
Model	52.09	6	8.68	3.47	0.0356
A	11.13	1	11.13	4.45	0.0587
B	11.30	1	11.30	4.52	0.0570
C	2.76	1	2.76	1.10	0.3159
AB	3.73	1	3.73	1.49	0.2479
AC	2.98	1	2.98	1.19	0.2981
BC	15.75	1	15.75	6.30	0.0290
Residual	27.52	11	2.50	-	-
Total	79.61	17	-	-	-

**Table 8:** Coefficient estimates demonstrating the response change per unit change in factor value when the remaining factors remain constant. The intercept is the average response of the runs. The coefficients are normalized by the average. For orthogonal factors the VIFs are 1; VIFs > 1 are the indications of multi-collinearity.

Factor	Coefficient Estimate	df	Standard Error	95% CI Low	95% CI High	VIF
Intercept	−11.57	1	0.3766	−12.40	−10.74	
A	0.9689	1	0.4595	−0.0424	1.98	1.02
B	0.9543	1	0.4490	−0.0339	1.94	1.00
C	0.3957	1	0.3766	−0.4332	1.22	1.02
AB	−0.6684	1	0.5477	−1.87	0.5371	1.02
AC	0.4974	1	0.4554	−0.5050	1.50	1.00
BC	−1.12	1	0.4484	−2.11	−0.1381	1.02

**Table 9:** Calculated mechanical properties of IBU.

Mechanical Property	Value
Bulk modulus (GPa)	7.74
Shear modulus (GPa)	1.66
Compressibility (GPa^−1^)	0.13
Young modulus (GPa)	7.97
Ex	6.19
Ey	7.34
Ez	8.69
Universal anisotropy index	1.97

**Table 10:** Wet media milling equipment design specifications supporting 12 tons annual production of IBU.

Parameter		Unit	Qa	Parameter		Unit	Qa
Planetary Ball Mill Design Specifications				Ball Milling Media			
Disk rotation speed	Ns	rpm	240	Diameter	d_b	m	0.008
Bowl rotation speed	Nb	rpm	250	Number	Nbw	-	5.385
Radius of Sun disk	R	m	0.3	Volume	Vb	cm^3^	0.2680
Mill jar diameter	d	m	0.2118	Weight		kg	0.0015
Mill jar height	h	m	0.1365	Weight total	w	kg	8.199
Mill jar volume	V	cm^3^	4812.38	IBU mass ratio	-	-	38.55
Process Parameters of Comminution							
Weight of feed	m	kg	0.2127	Absolute velocity	Vb	m/s	6.996
Mechanical efficiency	-	-	0.8	Kinetic energy	Kb	joule/hit	0.037
Specification of Energy				Miscellaneous			
Energy per Weight	E	J × h/g	12,129	Frequency	f	s^−1^	2.387
Specific Power	-	kJ/kg	43,666	Total frequency	ftot	s^−1^	12,855.7
-	-	-	-	Power consumption	P	Watt	449.53
-	-	-	-	Process time	t	h	1

**Table 11:** Particle size distribution, air inlet and outlet temperature, IBU and gas moisture content, air solid and gas moisture feed are presented. The predicted operational space is pointed out inside the frame covering all process parameters of the industrial SD.

T_in_	PSD10	PSD15	PSD16	PSD18	MOIST	TEMP	AIR	SOLID	GAS
							MOIST	MOIST	MOIST
°C	0.0045 mμ	0.3 mμ	0.75 mμ	2.5 mμ	KG/KG DRY	C	KG/HR	KG/HR	KG/KG DRY
84	2.43 × 10^−6^	0.016	0.051	0.230	40.08	48.5	5.21	0.000851	0.0149
88	2.89 × 10^−6^	0.019	0.056	0.241	34.63	49.8	5.56	0.000851	0.0159
92	3.82 × 10^−6^	0.022	0.065	0.251	29.04	51.0	5.92	0.000851	0.0170
96	7.21 × 10^−6^	0.029	0.079	0.255	23.31	52.2	6.28	0.000851	0.0180
100	8.49 × 10^−6^	0.035	0.093	0.269	17.45	53.4	6.66	0.000851	0.0191
104	0.00110	0.500	0.324	0.002	2.59 × 10^−8^	50.3	7.77	1.61 × 10^−9^	0.0223
108	0.00110	0.500	0.324	0.002	6.27 × 10^−8^	53.3	7.77	4 × 10^−9^	0.0223
112	0.00110	0.500	0.324	0.002	5.13 × 10^−8^	56.3	7.77	3.27 × 10^−9^	0.0223
116	0.00110	0.500	0.324	0.002	4.79 × 10^−8^	59.3	7.77	3.06 × 10^−9^	0.0223
120	0.00110	0.500	0.324	0.002	3.53 × 10^−8^	62.4	7.77	2.25 × 10^−9^	0.0223
124	0.00110	0.500	0.324	0.002	5.03 × 10^−8^	65.4	7.77	3.21 × 10^−9^	0.0223
128	0.00110	0.500	0.324	0.002	7.36 × 10^−8^	68.4	7.77	4.7 × 10^−9^	0.0223
132	0.00110	0.500	0.324	0.002	5.18 × 10^−8^	71.4	7.77	3.31 × 10^−9^	0.0223
136	0.00110	0.500	0.324	0.002	6.18 × 10^−8^	74.5	7.77	3.94 × 10^−9^	0.0223

## Supplementary Materials

The following are available online at https://www.mdpi.com/1999-4923/12/10/969/s1.
