# Supplementary material for: Overcoming the Solubility Barrier of Ibuprofen by the Rational Process Design of a Nanocrystal Formulation
Source: Pharmaceutics. 2020 Oct 14;12(10):969. doi: 10.3390/pharmaceutics12100969 (PMC7602516; doi:10.3390/pharmaceutics12100969)
Supplement: Supplementary file 1 [file pharmaceutics-12-00969-s001.pdf]

pharmaceutics

MDPI

SupplementaryMaterials

Overcoming the Solubility Barrier of Ibuprofen by the Rational Process Design of a Nanocrystal Formulation

Andreas Ouranidis *, Nikos Gkampelis, Elisavet Vardaka, Anna Karagianni, Dimitrios Tsiptsios, Ioannis Nikolakakis and Kyriakos Kachrimanis *

Process Design

S1. Modeling of the WMM process for Power and Work estimation

To model the nanogrinding process of IBU in continuous operation the Rittinger comminution law was employed to approximate the available energy, residence time, the geometry and dimensions of the balls and jar and the properties of the milling media (elastoplastic and friction coefficient), angular velocities and charge friction. Power and work were calculated for the characteristic desirable product IBU particle size d_{80}. The power requirement (in Watt) was given by:

$$\mathrm{POWER} = \frac{0.01\,(\sqrt{\mathrm{X_F}}-\sqrt{\mathrm{X_P}})\times \mathrm{BWI}\times \mathrm{FLOWT}}{\sqrt{\mathrm{X_P}\times \mathrm{X_F}}}. \quad (1)$$

where X_F is the diameter of > 80% of feed particle mass (m), X_P the diameter of > 80% of product particle mass (m), *BWI* the Bond work index and *FLOWT* the total solids mass flow rate (kg/s). The result was adjusted to the breakage function:

$$\mathrm{POWER_{adj}} = \mathrm{POWER} \times \left(\frac{\mathrm{X_P} + 10.6\times 10^{-6}}{1.145\mathrm{X_P}}\right). \quad (2)$$

The Bond equation was implemented to estimate the work consumed for the comminution process as,

$$\mathrm{E} = \mathrm{E_i}\,\frac{(\sqrt{\mathrm{d_F}}) - (\sqrt{\mathrm{d_P}})}{\mathrm{d_F}} \times \sqrt{\frac{100}{\mathrm{d_p}}} \quad (3)$$

where E is the work required to reduce a unit weight of feed with *80%* passing a diameter $\boldsymbol{d_F}$ microns to a product with *80%* passing a diameter $\boldsymbol{d_p}$ microns, E_i the Bond work index, i.e. the work that is required to reduce a unit weight from a theoretical infinite size to *80%* passing a diameter of 100 micrometers in *(kWh/ton)*. E_i is corrected according to King's efficiency factors as follows:

$F_1 = 1.3$ if the mill is used in an open circuit the Bond Work index (4)

$F_3 = (\frac{2.44}{D})^{0.2}$ fit for diameters in micro to nano scale (5)

$F_5 = 1 + \frac{0.013}{\frac{d_{80,I}}{d_{80,P}} - 1.35}$ if size reduction ratio $d_{80,I}\ /\ d_{80,P} > 3$ (6)

$$\mathrm{E_i} = \mathrm{E_{i,0}F_1F_3F_5} \quad (7)$$

The total relative impact energy of the system, offers an upper bound estimate of the amount of energy available for grinding i.e. the mill charge.

S2. Distribution Functions

The RRSB (Rosin—Rammler—Sperling—Bennet) or Weibull distribution which presents the cumulative fraction of solid material in particles smaller than or equal to a given diameter, was implemented for our model;

$$Q(d) = 1 - \exp\left\{-\left(\frac{d}{d_{63}}\right)^n\right\} \quad (8)$$

where n is the dispersion parameter determined by the distribution steepness χ by the equation

$$n = \frac{\ln\left(\frac{ln\,0.75}{ln 0.25}\right)}{ln\chi} \quad (9)$$

While $\mathbf{d_{63}}$ was calculated based on the specified median value according to the equation

$$d_{63} = \frac{d_{50}}{(\ln 2)^{1/n}} \quad (1)$$

The particle size distribution was then normalized by the value of the largest class interval (if Q value ranges between 0.95 to 1.0, hence RRSB is a function asymptotic to 1.0). The mechanical efficiency of grindability, which specifies the fraction of the required power that contributes to crushing while the rest is converted to heat and added to the enthalpy of the outlet stream, was also considered.

S3. Modeling of the SD process

Ideally SD would produce a single size droplet, yielding a single particle size product. In practice different sizes of droplets form, so the distribution of droplet size (DSD) was calculated utilizing hollow cone nozzle geometry techniques i.e. the approach of Walzel was implemented to find the Sauter mean diameter (d_{SM}) of the droplet size distribution;

$$d_{SM} = 1.6 D_A \, We_{hc}{}^{-\frac{1}{3}} \, \kappa^{0.4} \, (1 + 5\,Oh)^{\frac{3}{2}} \quad (2)$$

D_A being the nozzle outlet number while the Ohnesorge number (Oh) is related to the stability of the jet leaving the nozzle and is quantified as

$$Oh = \frac{n_{liq}}{\sqrt{\sigma_{liq}\rho_{liq}{}^{d}}} \quad (3)$$

where $n_{liq,}$ σ_{liq} and ρ_{liq} are the viscocity, surface tension and density of the liquid, respectively. The Weber number of a hollow cone nozzle was also calculated as

$$We_{hc} = \frac{D_A \, \rho_{liq} \, v_a{}^2}{\sigma_{liq}} \quad (4)$$

σ_{liq} and ρ_{liq} being the surface tension and density of the liquid respectfully, v_a is the exit velocity from the nozzle calculated of the potential velocity in the absence of losses, deriving from the Bernouli equation for incompressible fluids ignoring the inlet velocity. The actual exit velocity is then corrected using the rate coefficient φ as follows:

$$v_{A} = \varphi\, v_{pot} \tag{5}$$

where

$$v_{pot} = \sqrt{\frac{2\Delta_p}{\rho_{liq}}} \tag{6}$$

where Δ_p is the pressure drop over the nozzle and κ is the number of segments given by

$$\kappa = \frac{\mu}{2\pi\varphi \sin\left(\frac{\Theta}{2}\right)} \tag{7}$$

where

$$\mu = \frac{4\,\dot{V}_{liq}}{\pi\, D_A{}^2 v_{pot}}, \dot{V}_{liq} \tag{8}$$

Stands for the volumetric flow rate per nozzle and Θ is the full spray angle. The drying medium, flowing in at rate $\dot{m}_{gas,in}$ is pre heated at duty $\dot{Q}_{in}$. The solids and liquid mixture enter the spray tower at rate $\dot{m}_{s,in}$. The outlet material flows at rate $\dot{m}_{gas,out}$ and $\dot{m}_{s,out}$. The amount of $\dot{Q}_{loss}$ is expended to the environment. The overall mass balance is calculated by the equation

$$\dot{m}_{gas,in} + \dot{m}_{s,in} = \dot{m}_{gas,out} + \dot{m}_{s,out.} \tag{9}$$

The feed stream of the gas, *Y*, is given by the ratio of the mass flow of moisture dried in the exiting gas to the mass flow of dry gas,

$$Y = \frac{\dot{m}_{gas,w}}{\dot{m}_{gas,dry}}. \tag{19}$$

The loading of the solid, *X* is given by the ratio of the mass flow of moisture in the exiting solids to the mass flow of solids excluding all moisture,

$$X = \frac{\dot{m}_{s,w}}{\dot{m}_{s,dry}}. \tag{10}$$

The mass balance of the moisture is considered,

$$Y_{in}\, \dot{m}_{gas,dry} + X_{in}\, \dot{m}_{s,dry} = Y_{out}\, \dot{m}_{gas,dry} + X_{out}\, \dot{m}_{s,dry}\,, \tag{11}$$

while the flow of dry gas and dry solid is considered constant; opposite to the flow of the water. The enthalpy of inlet and outlet material flows, the heat for pre heating the gas and the heat lost constitute the enthalpy balance equation,

$$0 = \dot{H}_{gas,in} + \dot{H}_{s,in} + \dot{Q}_{in} - \dot{H}_{gas,out} - \dot{H}_{s,out} - \dot{Q}_{loss.} \tag{12}$$

The enthalpy of moist gas was calculated from the flows of dry gas and moisture, the heat capacities of dry gas $C_{p,gas,dry}$ and moisture $C_{p,w}$ and the enthalpy of vaporization at the reference state, $\Delta H_{v,0.}$ Thus the enthalpies of incoming and exiting gas will be described as,

$$\dot{H}_{gas,in} = \dot{m}_{gas,dry}\, ((\, C_{p,gas,dry} + Y_{1Cp,w})\, T_{gas,in} + Y_{in}\Delta H_{v,0}) \tag{13}$$

$$\dot{H}_{gas,out} = \dot{m}_{gas,dry} \left(\left(C_{p,gas,dry} + Y_{1Cp,w} \right) T_{gas,out} + Y_{out} \Delta H_{v,0} \right) \quad (14)$$

The enthalpy of the solid / liquid mixture is calculated using the solid loading and heat capacity as,

$$\dot{H}_{s,in} = \dot{m}_{s,dry} \left(C_{p,s,dry} + X_{inCp,w} \right) T_{gas,in} \quad (15)$$

$$\dot{H}_{s,out} = \dot{m}_{s,dry} \left(C_{p,s,dry} + X_{outCp,w} \right) T_{gas,out} \quad (16)$$

S3.1. SD Temperature / Feed / Particle Velocity Model

The drying process was modeled by a digital twin governed by differential equations integrated with respect to z axis, i.e. by consensus meaning the vertical Cartesian axis within the spay dryer. Regarding the particles, a differential equation is appointed for each class i, for each temperature T, of loading X and velocity v. These shall depend on the convective heat flux $Q_{conv,i}$, the velocities v of the particles and gas, the mass m and mass flux $\dot{m}$ of vapor ad solids, total liquid and solid-free moisture (w), the heat of vaporization Δh_v and finally the relative velocity

$$v_{rel} = v_{gas} - v_{liq} \quad (17)$$

$$\frac{dT_{p,i}}{d_z} = \frac{Q_{conv,i} - \Delta h_v \ m_{v,i}}{v_{gas} \ \dot{m}_{s,i} \left(c_{s,i} + c_{liq,i} \dot{X_i} \right)} \quad (18)$$

$$\frac{dX_i}{dz} = \frac{- \dot{m}_{v,i}}{v_{gas} \ \dot{m}_{s,i}} \quad (29)$$

$$\frac{dv_{p,i}}{dz} = \frac{1}{v_{p,i}} \left(g - \frac{3}{4} \ c_{w,i} \frac{\rho_{gas} \ v^2{}_{rel}}{\rho_p \ d_p} \right) \quad (19)$$

Regarding the gas, the interaction effects with the particle classes are summoned, yielding a differential equation for temperature T and one for feed Y. These shall depend on the number of particles n in each class, the mass of dry gas and several common parameters recognized above in the particle equations

$$\frac{dT_{gas}}{d_z} = \frac{\sum_i \left(\dot{n}_i m_{v,i} \left(T_{p,i} \, c_{liq} \ + \Delta h_v \ \left(\ T_{p,i} \right) - c_{p,d} T_1 \ - \ n_i \dot{Q}_{conv,i} \right) \right.}{\dot{m}_{gas,dry} \ v_{gas} \left(c_{p,gas} \ + c_{p,d} Y \right)} \quad (20)$$

The inlet temperature and feed of the solid / liquid phase are set as the initial conditions of the integration. The mass of the solids per particle per given size and also the total particle population are treated as constant (avoiding interactions between drops), based on the initial conditions of saturated droplets:

$$m_{s,p} \ = \frac{{}^1\!/_6 \, \pi \, d_p^3 \quad \rho_p}{1+X} \text{ and } \dot{n} = \frac{\dot{m}}{(1+X) m_{s,p}} \quad (21)$$

S3.2. Spray Dryer Evaporation Model

The evaporation rate was calculated as a normalized evaporation rate, where 1 refers to the initial drying rate of the saturated droplet as,

$$v = \frac{\dot{M}}{\dot{M}_0} = 2 \, F \, \frac{X_{wet}}{X_{crit,wet}} - (2 \, F - 1) \left(\frac{X_{wet}}{X_{crit,wet}} \right)^2 \quad (22)$$

pharmaceutics

MDPI

where $\dot{M}$ and $\dot{M}_0$ are the normalized and the initial evaporation rate of saturated droplet, respectfully. The rate is corrected by a shape factor F which varying from 0 to 1 , the critical moisture content $X_{crit,wet}$. , the residual moisture content X_{wet} .

S3.3. SD Particle Formation Model

The particle number was modeled separately for the different phases of drying; until critical moisture content reached, it is considered as spherical liquid containing fixed quantity of solid i.e. ideal shrinkage

$$d_p = \left[m_s\left(\frac{1}{\rho_s}\right) + \left(\frac{X_{dry}}{\rho_l}\right)\frac{6}{\pi}\right]^{1/3}, \text{ for } X > X_{crit} \quad (23)$$

Since the particle model of inflation of the particle is not considered it was assumed that the particle diameter remains constant after the point where the critical moisture content is met

$$d_{crit=}\left[m_s\left(\frac{1}{\rho_s}\right) + \left(\frac{X_{crit}}{\rho_l}\right)\frac{6}{\pi}\right]^{1/3} \quad (24)$$
